# Activation of Toll-Like Receptors by Live Gram-Negative Bacterial Pathogens Reveals Mitigation of TLR4 Responses and Activation of TLR5 by Flagella

**DOI:** 10.3389/fcimb.2021.745325

**Published:** 2021-11-23

**Authors:** Kei Amemiya, Jennifer L. Dankmeyer, Robert C. Bernhards, David P. Fetterer, David M. Waag, Patricia L. Worsham, David DeShazer

**Affiliations:** ^1^ Bacteriology Division, United States Army Medical Research Institute of Infectious Diseases, Fort Detrick, Frederick, MD, United States; ^2^ Edgewood Chemical Biological Centre, Aberdeen Proving Ground, Edgewood, MD, United States

**Keywords:** TLRs, gram-negative, live pathogens, innate immunity, TLR4 suppression, TLR5 activation, flagella

## Abstract

Successful bacterial pathogens have evolved to avoid activating an innate immune system in the host that responds to the pathogen through distinct Toll-like receptors (TLRs). The general class of biochemical components that activate TLRs has been studied extensively, but less is known about how TLRs interact with the class of compounds that are still associated with the live pathogen. Accordingly, we examined the activation of surface assembled TLR 2, 4, and 5 with live Tier 1 Gram-negative pathogens that included *Yersinia pestis* (plague), *Burkholderia mallei* (glanders), *Burkholderia pseudomallei* (melioidosis), and *Francisella tularensis* (tularemia). We found that *Y. pestis* CO92 grown at 28°C activated TLR2 and TLR4, but at 37°C the pathogen activated primarily TLR2. Although *B. mallei* and *B. pseudomallei* are genetically related, the former microorganism activated predominately TLR4, while the latter activated predominately TLR2. The capsule of wild-type *B. pseudomallei* 1026b was found to mitigate the activation of TLR2 and TLR4 when compared to a capsule mutant. Live *F. tularensis* (Ft) Schu S4 did not activate TLR2 or 4, although the less virulent Ft LVS and *F. novicida* activated only TLR2. *B. pseudomallei* purified flagellin or flagella attached to the microorganism activated TLR5. Activation of TLR5 was abolished by an antibody to TLR5, or a mutation of *fli*C, or elimination of the pathogen by filtration. In conclusion, we have uncovered new properties of the Gram-negative pathogens, and their interaction with TLRs of the host. Further studies are needed to include other microorganism to extend our observations with their interaction with TLRs, and to the possibility of leading to new efforts in therapeutics against these pathogens.

## 1 Introduction

The initial interaction of a pathogen with the host is a critical time for both the pathogen and the host. This early encounter with the pathogen is so vital for survival of the host that through the course of evolution invertebrates, vertebrates, and plants have developed an inheritable innate immune system to counteract pathogens. The innate immune system is one arm of the host’s defense immune system that coupled with the acquired immune system protects the mammalian hosts from an array of different pathogens. The innate immune system responds to an assortment of ligands associated with the pathogen that have been classified as pathogen associated molecular patterns (PAMPs) that require special pathogen recognition receptors (PRRs) for detection (Janeway and Medzhitov, 2002). Subsequently, the acquired immune response involves the host’s T- and B-cells that respond to signals produced by the innate immune system to direct the acquired immune response in a more pathogen-specific manner (Akira et al., 2006).

**Figure 1:**
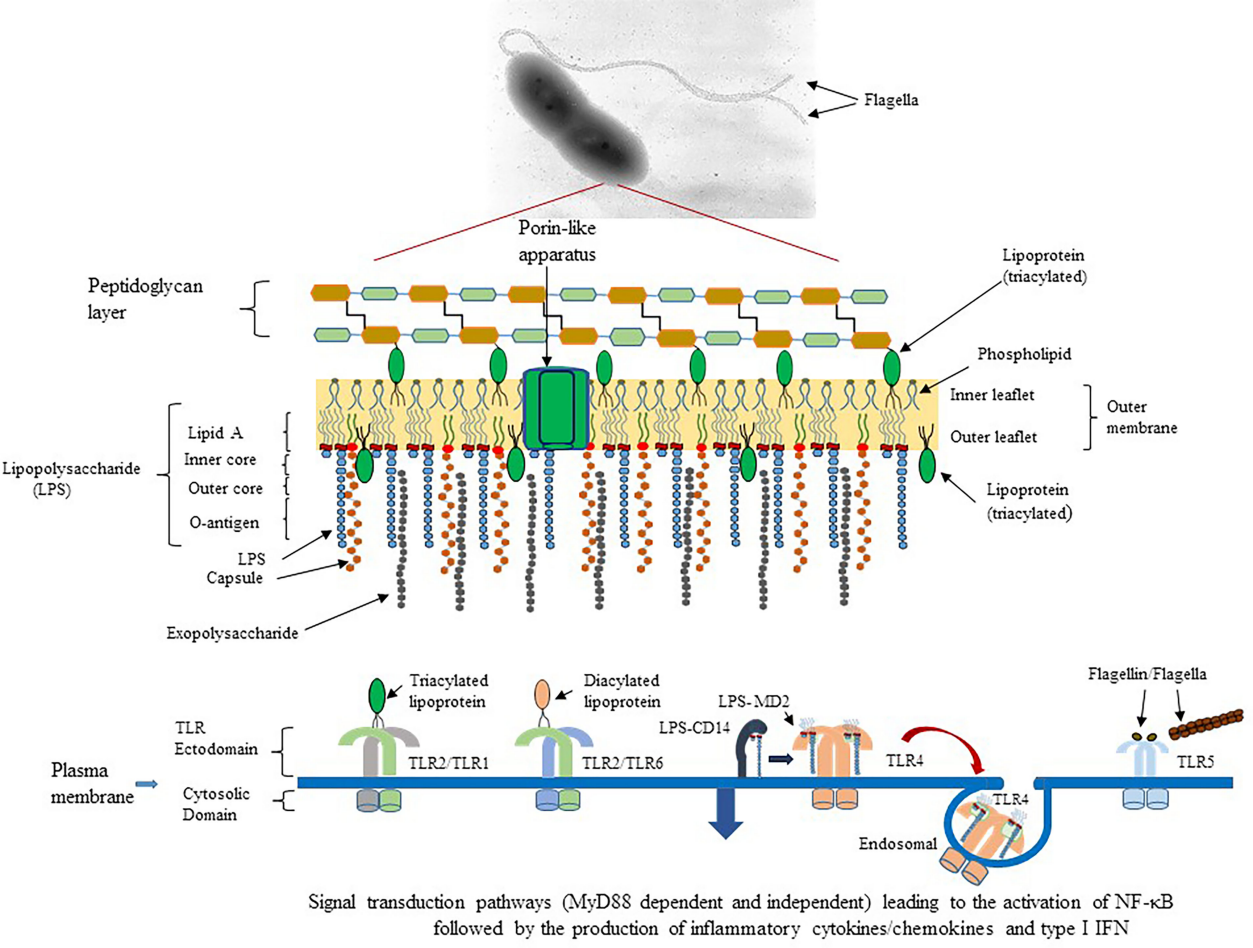
Schematic diagram of potential outer membrane components of Gram-negative pathogens that could interact with surface expressed TLR of the host’s innate immune system. An example of *B. pseudomallei* 1026b, a motile, human pathogen that causes melioidosis, and the presence of pathogen associated molecular patterns (PAMPs) or molecules that can activate TLR of the host is presented (Kawai and Akira, 2010). TLR2/TLR1 (heterodimer) could be activated by triacylated lipoproteins that are embedded in the inner and outer leaflet of the bacterial membrane. TLR2/TLR6 (heterodimer) can be activated by diacylated lipoproteins that are usually found in Gram-positive bacteria. TLR4 (homodimer) is activated by lipid A which is part of the LPS moiety found in the outer membrane of Gram-negative bacteria. LPS molecules are recognized by CD14 and transferred to the MD2-TLR4 complex before activation of TLR4. A subset of the LPS-MD-TLR4 complex is brought into the host cell by endocytosis, which is facilitated by CD14. TLR5 (homodimer) is activated by the flagellin molecule, which is the subunit of flagella. Activation requires at least one flagellin molecule per TLR5 molecule for optimal activation of the homodimer. In addition, TLR5 can be activated by flagella, which we show in the current report. All surfaced expressed TLRs transmit their activation signal across the host membrane into the cytoplasm and recruit the signal transduction protein MyD88 and TIRAP to transmit the signal leading to activation of NF-κB and expression of AP-1 to stimulate proinflammatory cytokine/chemokine production. However, the endosomal LPS-MD2-TLR4 complex recruits TRIF and TRAM to transmit the signal for TLR4 activation, which leads to the activation of NF-κB and induction of proinflammatory cytokines/chemokines and antimicrobial type I IFNs.

One family of PRRs is the Toll-like receptors (TLRs) of which humans have 10 members and mice have 12 (Takeuchi and Akira, 2010). The location that TLRs are assembled by the host cell reflects the type of ligand that it might encounter during the interaction with a pathogen. TLR1, TLR2, TLR4, TLR5, TLR6, and TLR11 are found on the surface of cells. TLR11 in mice recognizes uropathogenic bacteria (Zhang et al., 2004) or a profiling-like protein from the protozoan Toxoplasma gondii (Yarovinsky et al., 2005), but in humans, it is present as a pseudogene. On the other hand, TLR3, TLR7, TLR8, and TLR9 are expressed in intracellular endosomal compartments (Kawai and Akira, 2010). For the purpose of this present report, we are focusing on only the surface assembled TLRs (see **Figure 1**). In the former group, TLR1 combines with TLR2 (heterodimer) to recognize triacyl-lipopeptides, and the heterodimer TLR2/TLR6 recognizes diacyl-lipopeptides (Akira et al., 2006; Kang et al., 2009; Kurokawa et al., 2012; Nakayama et al., 2012). The TLR4 homodimer recognizes lipopolysaccharides (LPS) but may require other accessory factors, such as LPS binding protein (LBP), myeloid differentiation factor 2 (MD2), and CD14 for maximal activation (Poltorak et al., 1998; Shimazu et al., 1999; Akira et al., 2006). Unlike other TLRs assembled on the surface of the cell, a subset of TLR4-MD2-LPS complexes may be recruited into an endosomal compartment to activate an alternative signal transduction pathway for the induction of proinflammatory cytokines and type I interferons (IFNs) (Kagan et al., 2008). The homodimer TLR5 is activated by the bacterial flagellin subunit, which is the major subunit of the assembled bacterial flagella (Hayashi et al., 2001; Donnelly and Steiner, 2002; Smith et al., 2003).

Our interest has been focused on specific Gram-negative, bacterial pathogens (*Yersinia pestis, Burkholderia mallei, Burkholderia pseudomallei*, and *Francisella tularensis*) that are considered Tier 1 biological agents by the Centers for Disease Control and Prevention and are also Biosafety Level 3 (BSL3) biological agents. When the surface of these Gram-negative pathogens was examined, we saw an array of potential structures/organelles that protected or shielded the pathogen from unfriendly environments, for example, antimicrobial peptides or antibiotics or the host’s immune response. In **Figure 1** we show an example of potential components on the outer surface of the Gram-negative, motile pathogen *B. pseudomallei* 1026b, which is the etiological agent of melioidosis, that could interact with TLRs on the surface of host cells. One of the most abundant molecules is lipopolysaccharide (LPS), which has been estimated to cover three-quarters of the bacterial surface with approximately 1-2 X 10^6^ molecules (Whitfield and Trent, 2014). Lipid A is the endotoxin part of LPS and is the agonist for the host’s TLR4. The acylated Lipid A is embedded within the outer membrane leaflet and anchors the LPS molecule to the surface of the pathogen. Other structures that may be present are extracellular polysaccharides (EPS) that include a capsule and/or exopolysaccharide molecules. Both molecules protect the bacteria from adverse environmental conditions, from the host’s immune responses, such as opsonization/phagocytosis, and from complement mediated bacterial lysis (Whitfield, 2006; Warawa et al., 2009; Whitney and Howell, 2013; Schmid et al., 2015). Another molecule present is lipoprotein (Lpp) (Braun lipoprotein), which is also one of the most abundant proteins of the bacterial cell (~1 x 10^6^ molecules per cell) (Braun and Rehn, 1969; Li et al., 2014). Most Lpps are unbound or free (2/3) and a smaller fraction (1/3) is in the periplasm embedded in the inner leaflet of the OM, with some molecules linked to the peptidoglycan (PG) layer (bound portion) (Braun and Rehn, 1969; Inouye et al., 1972). Diacylated and triacylated lipoproteins are synthesized and inserted into the membrane of bacterial cells (Wilson and Bernstein, 2016). A subset of the unlinked or free Lpp molecules, however, is exported through the OM and embedded into the outer leaflet of the OM with the carboxy-terminal end exposed to the outer surface of the bacterial cell (Cowles et al., 2011; Kovacs-Simon et al., 2011; Nakayama et al., 2012; Wilson and Bernstein, 2016; Asmar and Collet, 2018). The presence of triacylated lipoproteins was once thought to be exclusive to Gram-negative bacteria, but they have also been found in some Gram-positive bacteria, which were reported to only synthesize diacyl lipoproteins (Kovacs-Simon et al., 2011; Kurokawa et al., 2012; Nakayama et al., 2012).

Another bacterial organelle associated with some bacteria is the flagellum that is involved in motility and chemotaxis, which leads to enhanced adhesion, biofilm formation, and virulence (Josenhans and Suerbaum, 2002; Galyov et al., 2010; Duan et al., 2013; Haiko and Westerlund-Wikstrom, 2013). The flagellin molecule, which is the major subunit of flagella, can be divided into 4 domains (D0, D1, D2, and D3) (Yonekura et al., 2003; Ramos et al., 2004; Beatson et al., 2006). The amino acids in domains D0 and D1 contain the most conserved sequences within the flagellin molecule (Yonekura et al., 2003; Ramos et al., 2004). The highly variable D2 and D3 domains are arranged into folded β-sheets and protrude outward from the assembled flagellum core. The highly conserved sequences in regions D0 and D1 of flagellin presents a protein signature that are the target for TLR5 (Hayashi et al., 2001). At least one region in the N-terminal D0 region and another in the C-terminal D1 region of flagellin are required for activation of TLR5 (Smith et al., 2003; Andersen-Nissen et al., 2005). Furthermore, for full activation of TLR5, the interaction of two TLR5 polypeptides (homodimer) and two flagellin subunits are required (Yoon et al., 2012; Ivicak-Kocjan et al., 2013; Forstneric et al., 2016).

Pathogenic bacteria have evolved methods to mitigate the protective innate inflammatory response of the host. One of the best examples is presented by the Gram-negative pathogen *Y. pestis*, the etiological agent of the zoonotic disease plague (Perry and Fetherston, 1997). During the transmission of *Y. pestis* and infection of the mammalian host, the pathogen is subjected to two different temperature conditions (Hinnebusch et al., 2017). While in a flea at 23-28°C, and while in the mammalian host at 37°C. Kawahara et al. (2002) reported that *Y. pestis* grown at 27°C produced a mixture of tri-, tetra-, penta-, and hexa-acylated lipid A molecules. However, when *Y. pestis* was grown at 37°C, only the lesser acylated lipid A forms were present. When they examined the biological activity of lipid A from *Y. pestis* grown at 27°C it was more active on human cells in inducing the production of TNF-α than lipid A from the pathogen grown at 37°C. The temperature dependent changes in acylated forms of lipid A were also seen in two other pathogenic *Yersinia* strains (*Y. pseudotuberculosis* and *Y. enterocolitica*) (Rebeil et al., 2004). To test the hypothesis that *Y. pestis* can alter lipid A biosynthesis to overcome the host innate immune response, Montminy et al. (2006) genetically modified *Y. pestis* to produce a more potent LPS. In this case, the *Y. pestis* strain with the more potent lipid A substantially activated the innate immune system compared to the wild-type pathogen and was cleared from the host more effectively than the wild-type strain in a mouse model of plague.

Although much is known about the molecular structures of PAMPs and TLRs, it is not clear how these two components interact with each other *in situ*. For example, when the mammalian host is first exposed to an aerosol of the pathogen, the pathogen may enter through the nasal cavity where it may proceed to infect the host. When and how the innate immune system is activated during this initial phase of infection is still unclear, and it may be confined to localized areas of the host depending on the number of pathogenic bacteria involved. Currently, it is speculated that TLRs are activated by PAMPs that are released or escaped from the pathogen during growth or eventually released in the presence of human serum (Tesh et al., 1986; Zhang et al., 1998; Hellman et al., 2000; Kitchens et al., 2000; Vesy et al., 2000; Matsuura, 2013). In previous innate immune studies with *Yersinia pestis* the bacterial lipid components were purified or bacterial cells inactivated before they were examined for their ability to activate TLRs (Kawahara et al., 2002; Rebeil et al., 2004; Montminy et al., 2006; Matsuura et al., 2010). Thus, we asked if we could detect changes in TLR2 and TLR4 activation by a live pathogen, such as *Y. pestis* CO92, when grown at 28°C (more potent lipid A) or at 37°C (less potent lipid A) that would reflect the modification of lipid A at the two temperatures as previously reported (Kawahara et al., 2002; Rebeil et al., 2004; Montminy et al., 2006). We also asked how other live Tier 1, Gram-negative pathogens, such as *Burkholderia mallei* (*Bm*), *Burkholderia pseudomallei* (*Bp*), and *Francisella tularensis* (*Ft*) Schu S4, would interact with specific PRRs on a mammalian cell. *B. mallei* is the etiological agent of glanders, which is a zoonotic disease usually associated with horses, donkeys, and mules, and is found in the Middle East, Africa, and Central and South Americas (Whitlock et al., 2007; Verma et al., 2014). *B. pseudomallei*, which causes melioidosis is an endemic disease of humans in South-east Asia and Northern Australia that may mimic other diseases, such as tuberculosis (Cheng and Currie, 2005; Wiersinga et al., 2012). *F. tularensis* is a small, highly infectious, Gram-negative, intracellular pathogen that causes tularemia (Ellis et al., 2002). *Ft* subspecies tularensis Schu S4 (Type A) is a highly virulent strain found primarily in North America, while the live vaccine strain (LVS) of *Ft* is an attenuated stain of the subspecies *holarctica* (Type B) can be found in Europe, the Far East, and North America. *Ft* subspecies *novicida* is another closely related strain of Ft but is less virulent in animal models. The present report is the result of our studies to assess the interaction of live Gram-negative pathogens with TLR2, 4, and 5, that are expressed on the surface of host cells.

## 2 Methods

### 2.1 Bacterial Strains, Growth Media and Conditions, and Reagents

Bacterial strains used in this study are listed in **Table 1** with growth media, incubation temperature, and length of incubation. Bacterial strains were stored at -80°C, and those considered to be Tier 1 Bioagents were stored and handled under Biosafety Level 3 (BSL3) laboratory conditions as regulated by the Center for Disease Control and Prevention (CDC) under the guidance of the Biosafety Division at USAMRIID. BSL3 bacterial strains that needed to be investigated under BSL2 conditions, for example for LPS extraction, were first grown and suspended in PBS, and then inactivated by irradiation, and checked for sterility under BSL3 conditions before they were removed from the BSL3 laboratory. Sheep blood and chocolate agar plates were obtained from Remel (ThermoFisher, Waltham, MA). Tryptone, Luria-Bertani Lennox broth, and agar were obtained from Difco (Becton Dickenson, Sparks, MD). Glycerol was obtained from Sigma-Alrich (St. Louis, MO). For each study, bacterial strains were streaked out on fresh plates from -80°C stocks, and initial bacterial suspensions were prepared from growth on plates in Dulbecco’s 1xPBS (Gibco, ThermoFisher). The optical density (OD_600_ nm) of bacterial suspensions was adjusted to an OD of 1.0 with a Genesys20 spectrophotometer (Fisher Scientific, Waltham, MA) with disposable 1.0 ml cuvettes (Bio-Rad, Richmond, CA) that resulted in the following bacterial cell density (from Hank Heine, USAMRIID): *Y. pestis* spp., 5.34 x 10^8^ colony forming units (cfu)/ml; *Burkholderia* spp., 5.0 x 10^8^ cfu/ml; *Francisella* spp., 3.89 x 10^10^ cfu/ml. Further dilutions were made with 1xPBS to obtain the desired bacterial cell number for each assay.

**Table 1:** Bacterial strains used in the present study.

Bacterial Strains	Human Pathogen	Source	Growth Medium^c^	Incubation Period (Temperature)
*Yersinia pestis* (Yp) CO92	+	Critical Reagent Program, USAMRIID	SBA	36-48 h (27/28 or 37°C)
Yp C12 (F1-, Δcapsule)	+	Critical Reagent Program, USAMRIID	"	"
Yp EV76 (vaccine strain)	–	Bret Purcell, USAMRIID	"	"
*Burkholderia mallei* FMH	+	Critical Reagent Program, USAMRIID	GTA, LB, SBA	36-48 h (37°C)
*Burkholderia pseudomallei* (Bp) K96243	+	Critical Reagent Program, USAMRIID^b^	GTA, LB, SBA	36-48 h (37°C)
Bp DDL3319 (Δ*fliC*)	+	This study	"	"
Bp 1106a	+	Critical Reagent Program, USAMRIID	"	"
Bp MSHR305	+	"	"	"
Bp MSHR668	+	"	"	"
Bp 406e	+	"	"	"
Bp MSHR5855	+	"	"	"
Bp MSHR5848	+	"	"	"
Bp MSHR5858	+	"	"	"
Bp HBPUB10134a	+	"	"	"
Bp MSHR10303a	+	"	"	"
Bp 1026b	+	"	"	"
Bp 1026b *(*Δ*wcb*R-*wcb*A , Δcapsule)	+	Herbert P. Schweizer, N. Arizona Univ., Az	"	"
*B. thailandensis* (Bt) E264	–	Critical Reagent Program, USAMRIID	GTA, LB, SBA	"
Bt DDI3196 (Δ*fli*C)	–	This study	"	"
Bt DDI3196+pMo168+*fli*C	–	This study	"	"
*Francisella tularensis* Schu S4	+	Critical Reagent Program, USAMRIID	Chocolate Agar	72 h (37°C)
*F. tularensis* LVS^a^	–	"	"	"
*F. novicida*	+/-	Todd Kijek, USAMRIID	"	"

a Formally F. tularensis holarctica.

b Country origin and clinical source of B. pseudomallei strains described in (Amemiya et al., 2020).

c SBA, sheep blood agar; GTA, 4%glycerol tryptone agar; LB, Luria-Bertani Lennox Broth agar.

Positive controls for TLR activation (InvivoGen, San Diego, CA) were heat-killed *Listeria monocytogenes* (HKLM) for TLR2 (TLR2/6, InvivoGen), ultrapure LPS from *E. coli* K12 for TLR4, and purified flagellin from *S. typhimurium* (*St*) for TLR5. Anti-TLR5 antibody (InvivoGen) was stored at -20°C and working stock stored at 4°C. When the anti-TLR5 monoclonal antibody was used, it was added to the HEK293 TLR5 cell at least 10 min prior to adding FliC or bacterial cells. Purified *Bp* K96243 FliC (or coupled His6-MBP-tev-*Bp* FliC) was prepared and analyzed by Bill Gillette of the Protein Expression Laboratory (National Cancer Institute, Frederick, MD) and stored at -80°C. Final *Bp* K96243 FliC purification preparations (5 µg) was analyzed on a 10.5 – 14% Criterion Tris-HCl gel, 26 well, 15 µl (Bio-Rad, Hercules, CA). All controls were diluted with endo-toxin free water and stored (-20°C) as recommended by the manufacturer, except working stocks of LPS were stored at 4°C.

### 2.2 Human Embryonic Kidney 293 Cells Expressing Human TLRs and TLR Activation

Activation of individual TLRs was monitored by HEK293 cells that were co-transfected with human TLRs 2, 4, or 5, and a gene for secreted embryonic alkaline phosphatase (SEAP) was obtained from InvivoGen. In addition, HEK293-hTLR4 cells were co-transfected with co-receptors CD14 and MD2. The SEAP gene for HEK293-hTLR4 cells was under the control of the interleukin (IL)-12 p40 minimal promoter with an enhancer region with multiple NF-κB and AP-1 binding sites. The SEAP gene for HEK293-hTLR2 and hTLR5 cells was under the control of the minimal IFN-β promoter with an enhancer containing multiple NF-κB and AP-1 sites. The HEK293 cells expressed low endogenous amounts of TLR1 and TLR6 (InvivoGen), hence, when TLR2 was activated it dimerized with either TLR1 when activated by triacylated lipoproteins or TLR6 when activated by diacylated lipoproteins (Akira et al., 2006; Kang et al., 2009; Kurokawa et al., 2012; Nakayama et al., 2012).

To prepare HEK293-TLR cells for activation studies, cells were grown and maintained as recommended by the manufacturer (InvivoGen). Briefly, cells were grown in T-75 flasks (Corning, Fisher Scientific) containing 25−50 ml of DMEM containing 4.5 g/L glucose, 2 mM glutamine, 100 U/ml penicillin, 50 µg/ml streptomycin, and 10% (v/v) heat-inactivated fetal calf serum (all from Gibco/ThermoFisher). Antibiotic stocks of Normocin and Selection (InvivoGen) were added as recommended. For HEK293-TLR5 cells blasticidin (30 µg/ml) and zeocin (100 µg/ml) (InvivoGen) were added with Normocin to the media. HEK293-TLR cells were incubated at 37°C with 5% CO_2_, and cells were passed at least twice weekly and were harvested after they reached ~65−85% confluency for TLR studies. Cells were prepared after rinsing twice with 10 ml of pre-warmed DPBS, and incubated at 37°C for 10 min with ~1.0−2.0 ml of DPBS. Cells were dislodged by tapping the flask against the palm of your hand and scraping off residual cells before collecting. Single cell suspensions were made by pipetting up and down with a 1.0 ml pipette. The cells were diluted 1/10 in DPBS and counted, and cell density adjusted to the following initial cell concentration in HEK-Blue Detection medium (InvivoGen): 2.5 x 10^5^ cells/ml for HEK293-TLR2, 2.0 x 10^5^ cells/ml for HEK293-TLR4, and 1.4 x 10^5^ cells/ml for HEK293-TLR5.

For TLR activation studies 180 µl of the HEK293-TLR adjusted cell suspension was added to each well of a flat-bottom, 96-well Immulon 2HB plate (ThermoFisher), and 20 µl of endotoxin-free water to negative media control wells, or 20 µl of TLR positive control, or 20 µl of test sample. When examining BSL3 microorganisms HEK293-TLR cells were loaded onto the 96-well plates under BSL2 laboratory conditions, and 96-well plates were transferred into BSL3 laboratories before adding bacterial cells and controls. All test samples and controls as indicated in Figure legends were added in triplicate wells. The plates were incubated for 16-20 h at 37°C with 5% CO_2_, and plates read at 630 nm in a BioTek Elx808 spectrophotometer (BioTek, Winooski, VT). The microorganisms used in the present study do not grow in the HEK-Blue Detection medium as determined by plating the inoculated sample after overnight incubation, microscopic examination of the culture, or changes in the color indicator (red to yellow) of the medium.

### 2.3 LPS Extract Preparation and Western-Blot Analysis

LPS extract was prepared from desired bacterial cells grown as listed in **Table 1**, and cell suspension adjusted to an OD_600_ of 1.0 with DPBS. Cells were inactivated by irradiation and checked for sterility and stored at -20°C until sterility was confirmed. A total of ~5.0 ml of inactivated cells, after thawing on ice, was centrifuged 1 ml at a time in one tube at 13,000 rpm for 10 min at room temperature and was stored at -20°C until use. The method of Yi and Hackett (2000) was used to extract LPS from bacterial cells. Briefly, the cell pellet was suspended in 200 µl of TRIzol (Invitrogen, ThermoFisher), and samples left at room temperature for 15 min. 170 µl of chloroform was added to each sample, vortexed, and left at room temperature for 10 min. After centrifugation at 12,000 x g for 10 min, the upper layer was removed and placed into a new tube. 100 µl of endotoxin-free water (Invitrogen) was added to the chloroform extract, sample vortexed, and incubated for 10 min at room temperature, and upper layer removed to another new tube. The water extraction was repeated 3 times, and all extractions were combined. The samples were dried overnight, and samples were suspended in 0.5−1.0 ml of 0.375 M MgCl_2_ in 95% ethanol. Samples were centrifuged at 12,000 x g for 15 min, and pellets suspended in 100 µl of endotoxin-free water. Whole *Bp* cells used for capsule analysis were washed 2X with cold PBS and cells were suspended in 100 µl of PBS. For western blot analysis of LPS extract or capsule, a 10−20% Novex Tricine gel (Invitrogen, ThermoFisher) was used (9 µl for LPS or 12 µl for capsule) as described previously (Shea et al., 2017) with a primary anti-*Bp* 1026b LPS monoclonal antibody (11G3-1, 1/2000 dilution) or anti-*Bp* capsule monoclonal antibody (AVA5, 1/5,000 dilution). For examination of *Ft* Schu S4 or Ft LVS LPS extract by western blot analysis (7.5 µl each sample) a 10−20% Tricine gel was used. A 1/1000 dilution of anti-*Ft* Schu S4 LPS monoclonal antibody (FB11, from Joel Bozue, USAMRIID) was used as the primary antibody. The secondary antibody (1/5,000) was a peroxidase-labeled goat anti-mouse IgG (KPL, SeraCare LifeSciences, Gaithersburg, MD), and western blots were developed with tetramethylbenzidine membrane peroxidase substrate (KPL).

### 2.4 Construction of *B. pseudomallei* DDL3319 and *B. thailandensis* DDI3196 *fli*C Mutants

Restriction enzymes (Roche-Sigma Aldrich, St. Louis, MO, USA, and New England BioLabs, Lpswich, MA, USA), Antarctic phosphatase (New England BioLabs), and T4 DNA ligase (Roche-Sigma Aldrich) were used according to the manufacturer’s instructions. The DNA fragments used in cloning procedures were excised from agarose (Difco, Becton Dickenson) gels and purified with a PureLink Quick gel extraction kit (Invitrogen, ThermoFisher). Bacterial genomic DNA (gDNA) was prepared from overnight LB broth (Difco, Becton Dickenson) cultures with the GenElute bacterial genomic DNA kit (Sigma-Aldrich). Plasmids were purified from overnight LB broth cultures by using the Wizard Plus SV miniprep DNA purification system (Promega, Madison, WI, USA).

In this study, we constructed in-frame deletion mutations in the *B. pseudomallei* K96243 (BpK) *fliC* gene (*BPSL3319*) and the *B. thailandensis* (*Bt*) E264 *fliC* gene (*BTH_I3196*). In order to construct DDL3319, BpK gDNA was PCR-amplified with BpfliC-up (5’-GCTAGCGCTCACCGAACGATCGACAC-3’) and BpfliC-dn (5’-GCTAGCTTTGCTGCTGCGTCGTGCTG-3’) using FailSafe™ PCR Enzyme Mix and 2X PreMix D (Lucigen, Middleton, WI, USA). The thermocycling parameters involved an initial gDNA denaturation at 97°C for 5 min followed by 30 cycles of denaturation, primer annealing and extension at 97°C for 30 sec, 55°C for 30 sec and 72°C for 2 min. The 1,522-bp PCR product was cloned into pCR2.1-TOPO (Invitrogen, ThermoFisher, Waltham, MA, USA) and transformed into One Shot TOP10 Chemically Competent *E. coli* (Invitrogen, ThermoFisher*)*. The resulting plasmid, pCR2.1-Bp*fliC*, was digested with *Nhe*I and the 1,522-bp product was separated on an agarose gel. The plasmid pMo130ΔNX (Burtnick et al., 2011) was digested with *Nhe*I, treated with Antarctic Phosphatase and separated on an agarose gel. The two *Nhe*I fragments were ligated with T4DNA Ligase and the recombinant plasmid was designated as pMo130-Bp*fliC*. This plasmid was digested with *Pst*I, separated on an agarose gel, the 681-bp fragment was removed and the plasmid was re-ligated with T4 DNA Ligase. The resulting plasmid, pMo130-ΔBp*fliC*, contained a 681-bp in-frame deletion of the BpK *fliC* gene.

Gene replacement experiments with BpK were performed as previously described (Hamad et al., 2009; Logue et al., 2009; Burtnick et al., 2011). Briefly, pMo130-ΔBp*fliC* was electroporated into *E. coli* S17-1 (Simon et al., 1989) (12.25 kV/cm) and conjugated with BpK for 8 h. The cells were resuspended in PBS, aliquots were spread onto LB agar plates containing 500 μg/ml kanamycin and 25 μg/ml polymyxin B and incubated at 37°C for 48 h. The optimal conditions for the resolution of the *sacB* constructs were found to be LB agar lacking NaCl and containing 10% (wt/vol) sucrose with incubation at 25°C for 3 days. Sucrose resistant colonies were picked to LB plates containing 0.3% agar to assess motility after incubation at 37°C for 24 h. A motility mutant, termed DDL3319, was further characterized by PCR using the primers flanking the deleted region of *fliC* (BpfliC-up and BpfliC-dn). As expected, the PCR product generated from DDL3319 was smaller than the product obtained from BpK (data not shown).

Construction of *Bt* DDI3196 was constructed in a similar fashion. *Bt* E264 gDNA was PCR-amplified with BtfliC-up (5’-GCTAGCGGGCCGAATCTCATCATCTC-3’) and BtfliC-dn (5’-GCTAGCTGACGGTGGACATCGGATAG-3’) and the 1,452-bp PCR product was cloned into pCR2.1-TOPO. The resulting plasmid, pCR2.1-Bt*fliC*, was digested with *NheI*, separated on an agarose gel, and the 1,452-bp fragment was cloned into pMo130ΔNX. The recombinant plasmid, termed pMo130-Bt*fliC*, was digested with *Pst*I and the 666-bp insert was removed and the plasmid was re-ligated. S17-1 (pMo130-ΔBt*fliC*) was conjugated with *Bt* E264 as described above and a motility mutant with a 666-bp in-frame deletion in the *fliC* gene was generated (DDI3196). We also generated a broad-host-range plasmid containing the *Bt* E264 *fliC* gene for complementation studies. Plasmid pCR2.1-Bt*fliC* was digested with *Xba*I and *Hin*dIII and the insert was cloned into the corresponding sites of pMo168 (Hamad et al., 2009). The resulting plasmid, pMo168-Bt*fliC*, was electroporated into S17-1 and conjugated to DDI3196 as described above.

### 2.5 Statistical Analysis

Values were log transformed prior to analysis, with results summarized as geometric mean and geometric standard error of the mean (SEM). Comparisons between groups were made by Welch’s *t*-test, applied to the log transformed values. The comparison between groups were based on a Welch’s *t*-test of the appropriate single degree of freedom contrast in the two-way ANOVA having factors corresponding to TLR2/4 and treatment group. Analysis was implemented in SAS^®^ version 9.4 (SAS institute Inc., Cary, NC). Results were considered significant when *P* < 0.05.

## 3 Results

### 3.1 Activation of TLR2 and 4 by Live Gram-Negative Pathogens

#### 3.1.1 *Y. pestis* Can Modulate The Activation of TLR2 and TLR4 Depending on the Growth Temperature

**Figure 2:**
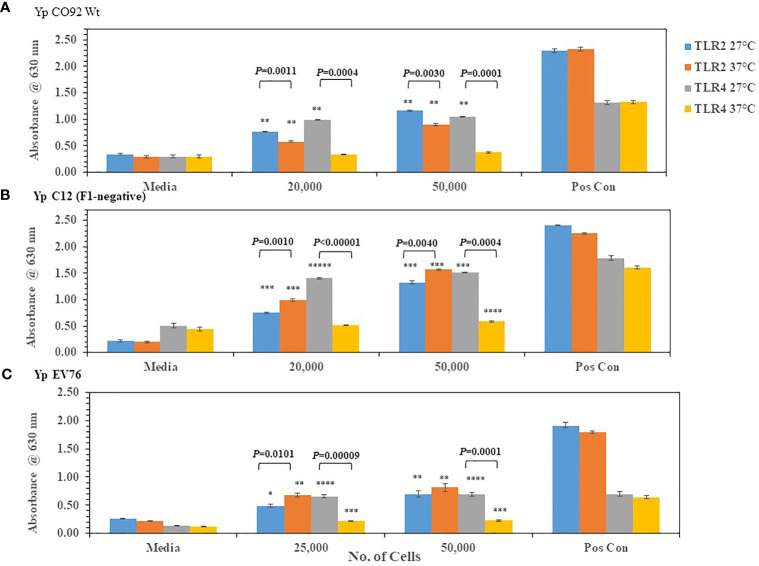
*Y. pestis* (*Yp*) can modulate the activation of TLR4 depending on its growth temperature. Strains of *Yp* were grown at 27−28°C or 37°C and suspended in PBS. Different amounts of cells were added to HEK293-TLR2 or -TLR4 cells to evaluate their ability to activate the TLRs. *Yp* strains used: **(A)** *Yp* CO92, wild-type; **(B)** *Yp* C12, a F1- or capsule negative strain; **(C)** *Yp* EV76, a vaccine strain;. Media controls contained only PBS, and positive controls contained either HKLM cells (2 x 10^6^ cells) for TLR2 or *E. coli* LPS (0.2 ng) for TLR4. All samples were analyzed in triplicate, and results represent one of two independent repeats. Results are presented as geometric mean with standard error of the mean. Significant differences between values at different temperatures are shown above the results. Significant values compared to media control are shown: *P ≤ 0.05; **P ≤ 0.01; ***P ≤ 0.001; ****P ≤ 0.0001; *****P ≤ 0.00001.

**Figure 3:**
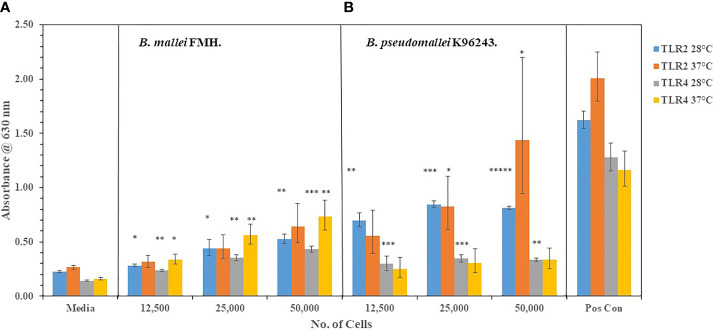
Neither *B. mallei* nor *B. pseudomallei* K96243 suppress activation of TLR2 or TLR4 at 37°C. *B. mallei* **(A)** and *B. pseudomallei* K96243 **(B)** were grown at 28°C or 37°C for ~48 h, and bacterial suspensions were diluted in PBS to inoculate HEK293-TLR2 or -TLR4 cells. Media controls contained only PBS rather than bacterial cells and positive controls contained either HKLM cells (2 x 10^6^ cells) for TLR2 or *E. coli* LPS (0.2 ng) for TLR4. All samples were analyzed in triplicate, and results represent one of two independent repeats. Samples were read at 630 nm after ~20 h of incubation. Results are presented as geometric mean with standard error of the mean. Significant values compared to media control are shown: *P ≤ 0.05; **P ≤ 0.0100; ***P ≤ 0.0010; *****P ≤ 0.00001.

We wanted to know if we could detect changes in TLR2/4 activation by the plague pathogen *Y. pestis* (*Yp*) depending on the temperature of growth that reflects changes in lipid A biosynthesis. We first grew *Yp* CO92 at 27-28°C (more potent lipid A) or at 37°C (less potent lipid A) on SBA plates. After two days of incubation, *Yp* CO92 cells grown at the two different temperatures were each suspended in PBS and cell concentration adjusted before adding the live pathogen to HEK293 cells expressing TLR2 or TLR4. The specificity of these TLR expressing HEK293 cells was previously tested by incubating the cells with the appropriate positive control antigen or another antigen. We saw a large amount of activation of the HEK293-TLR2 and -TLR4 cells with their respective positive control antigens (HKLM cells or *E. coli* K12 LPS, respectively) but no activation of TLR2 or TLR4 by the other control antigen, LPS or HKLM, respectively (see **Figure S1**). With *Yp* CO92 cells grown at 27°C we saw a significant amount of activation of both TLR2 and TLR4 compared to the media control (**Figure 2A**). But when *Y*p CO92 cells grown at 37°C were used as the inoculum, we saw an increase in activation of TLR2 with an increase in cell number; however, we did not see a simultaneous increase in TLR4 activation. In fact, there was a decrease in TLR4 activation by cells grown at the higher temperature compared to those grown at 27-28°C. We found this differential activation of TLR2 and 4 in other strains of *Yp* (C12 and EV76) we examined when they were grown at 27-28°C or 37°C (**Figures 2B, C**, respectively). In all these cases, there was a significant decrease in TLR4 activation at the higher temperature without a corresponding notable change in the level of TLR2 activation at the higher temperature. We did not note this differential change in TLR2 and TLR4 activation by *B. mallei* FMH or *B. pseudomallei* K96243 after a shift in growth temperature (**Figure 3**). However, activation of TLR4 at either temperature by *B*. *psuedomallei* K96243 appeared to be slightly suppressed compared to *B. mallei* FMH. Thus, our results with live, whole cells of *Yp* suggest that the changes we detected in TLR2 and TLR4 activation at the two different temperatures likely reflect the modifications in lipid A biosynthesis by the pathogen grown at 27-28°C or 37°C as previously reported (Kawahara et al., 2002; Rebeil et al., 2004; Montminy et al., 2006; Matsuura et al., 2010).

#### 3.1.2 The capsule of *B. pseudomallei* Mitigates TLR2 and TLR4 Activation

**Figure 4:**
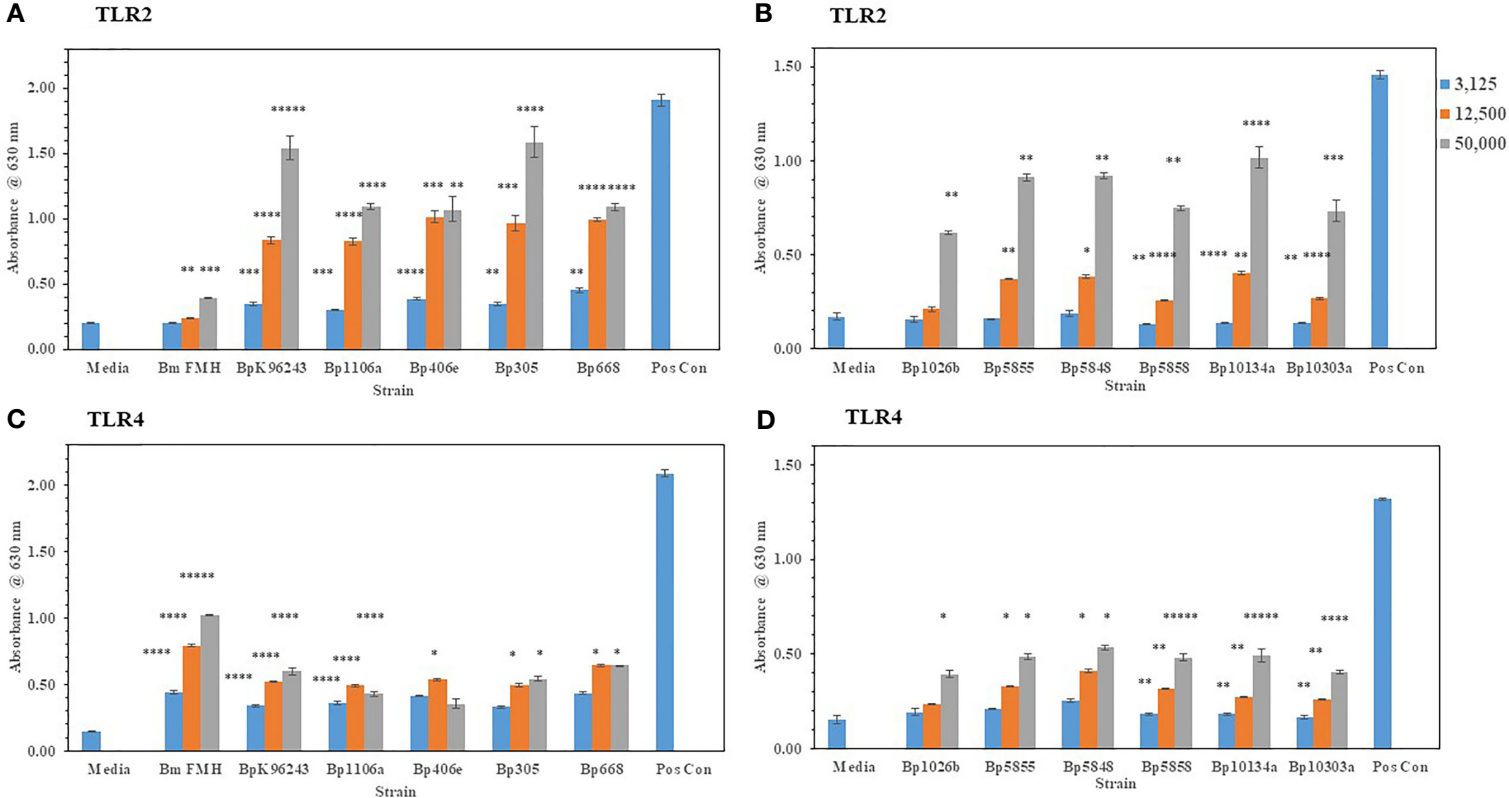
*B. mallei* (*Bm*) and *B. pseudomallei* (*Bp*) are closely related species but activate TLR2 and TLR4 differently. *Burkholderia* spp. were grown at 37°C for ~48 h, and bacterial suspensions were diluted in PBS to inoculate HEK293-TLR2 or -TLR4 cells. The following *Burkholderia* spp. were evaluated: **(A)** TLR2: *Bm* FMH, *Bp* K96243, *Bp* 1106a, *Bp* 406e, *Bp* MSHR305, and *Bp* MSHR668; **(B)** TLR2: *Bp* 1026b, *Bp* MSHR 5855, *Bp* MSHR5848, *Bp* MSHR5858, *Bp* HBPUB10134a, and *Bp* HBPUB10303a. **(C, D)** are TLR4 incubated with the same strains as listed for **(A, B)**, respectively. Media controls contained only PBS rather than bacterial cells and positive controls contained either HKLM cells (2 x 10^6^ cells) for TLR2 or *E. coli* LPS (0.2 ng) for TLR4. All samples were analyzed in triplicate, and results represent one of two independent repeats. Results are presented as geometric mean with standard error of the mean. Significant values compared to media control are shown: *P ≤ 0.05; **P ≤ 0.01; ***P ≤ 0.001; ****P ≤ 0.0001; *****P ≤ 0.00001.

We wanted to extent our examination of the interaction of live, Gram-negative pathogens with TLR2 or TLR4 with two other Gram-negative, facultative intracellular pathogens. They were *B. mallei* (*Bm*) and *B. pseudomallei* (*Bp*), which are closely related species but cause widely different diseases: glanders and melioidosis, respectively. *Bm* FMH and *Bp* strains were grown for 48 h and bacterial suspensions were made to inoculate HEK293- TLR2 or -TLR4 cells. As shown in **Figures 4A, C**, *Bm* FMH activated both TLR2 and TLR4, but we saw a significant increase in activation of TLR4 compared to the media control at all bacterial cell concentrations examined. Activation of TLR2 by *Bm* FMH was not as prominent as that of TLR4, although it was still higher than the media control sample. In contrast, all *Bp* strains examined activated TLR2 more than TLR4 (**Figures 4A–D**). In many cases at the lower (3,125 cells) and median (12,500 cells) bacterial cell concentrations, both TLRs appeared to be activated equally well and were measurably above the media control. But at the highest cell number (50,000 cells) examined TLR2 was the predominate TLR activated, and at the same time we could detect a low but significant increase in amounts of TLR4 activated. Thus, although these two Gram-negative pathogens are closely related (Nierman et al., 2004), the major TLR that was activated by live cells was TLR4 by *Bm* FMH and TLR2 by *Bp* strains.

**Figure 5:**
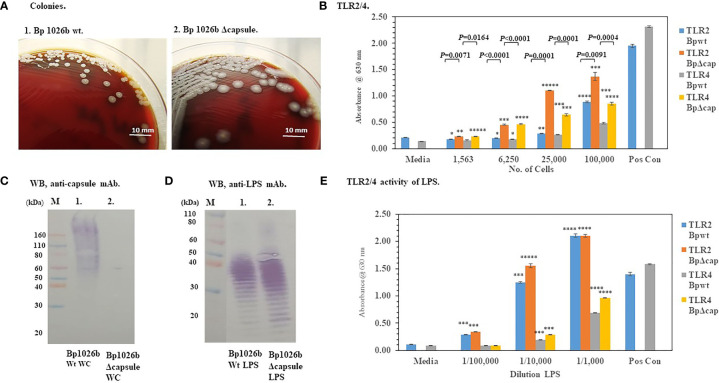
The capsule of *Bp* 1026b mitigates the activation of TLR2 and TLR4. *Bp* 1026b (Wt) and *Bp* 1026b (Δ*wcb*R-*wcb*A, Δcapsule mutant) were grown for ~48 h on SBA at 37°C. **(A)** Colony morphology of Wt and Δcapsule mutant. **(B)** Bacterial cell suspension were made in PBS and used to inoculate HEK293-TLR2 or -TLR4 cells and activation of TLRs measured after 18 h. Samples were performed in triplicate, and results represent one of two repeats. Significant differences in activity between Wt and capsule mutant are shown above the results. **(C)** Western blot (WB) analysis of whole-cells (WC) of *Bp* 1026b Wt and *Bp* 1026b Δcapsule mutant probed with an anti-capsule monoclonal antibody (mAb) (AVA5). **(D)** WB analysis of LPS extract from *Bp* 1026b Wt and *Bp* 1026b Δcapsule mutant probed with an anti-LPS mAb (11G3-1). **(E)** Activation of TLR2 or TLR4 by LPS extract from *Bp* 1026b Wt and *Bp* 1026b Δcapsule mutant. Samples were analyzed in triplicate and results represent one of two independent repeats. Results of TLR activation **(B, E)** are presented as geometric mean with standard error of the mean. Media controls were PBS, and positive controls (Pos Con) were HKLM (2 x 10^6^ cells) for TLR2, and *E. coli* LPS (0.2 ng) for TLR4. Significant values compared to media control are shown: *P ≤ 0.05; **P ≤ 0.01; ***P ≤ 0.001; ****P ≤ 0.0001; *****P ≤ 0.00001.

Because the capsule surrounds the bacteria with a protective layer we wondered if the presence of the capsule of *Bp* effected the activation of TLR2 or TLR4 (see **Figure 1**). **Figure 5A** shows that the absence of the capsule of *Bp* 1026b (Δ*wcb*R-*wcb*A) altered the morphology of the colonies of the bacteria after 48 h of growth compared to the wild-type strain. The wild-type strain was round (2−3 mm), grayish-white, wrinkled-edge colonies with small raised centers, whereas the capsule mutant colonies were larger (3−4 mm), grayish-white, and flatter. The absence of the capsule was further confirmed by a western blot analysis of whole-cells of the wild-type and capsule mutant probed with an anti-capsule monoclonal antibody (**Figure 5C**, lane 1 and 2, respectively). In **Figure 5C**, lane 1 of the western blot, we saw the presence of capsule material present in whole-cells of the wild-type *Bp* 1026a, but in **Figure 5C**, lane 2 of the western blot that contained whole-cells of the capsule mutant, there was no positive staining material present. When HEK293-TLR2 or -TLR4 cells were inoculated with the wild-type *Bp* 1026b or the *Bp*1026b capsule mutant, we saw a higher activation of TLR2 than TLR4 at the highest bacterial concentration examined that was similar to the pattern seen previously (**Figure 5B** compared to **Figures 4B, D**). However, the *Bp* 1026b capsule mutant activated significantly more TLR2 (*P* ≤ 0.0091−0.0001) and TLR4 (*P* =0.0164-<0.0001−0.0001) than the wild-type strain at equivalent cell numbers. We further examined the LPS extract of the wild-type and capsule mutant to show that there were no apparent changes in the molecular pattern (**Figure 5D**) and activity (**Figure 5E**) of the LPS extract of the capsule mutant compared to the wild-type strain. Thus, the bacterial capsule of *Bp* appears to mitigate the activation of the host’s innate immune response to the presence of the pathogen.

#### 3.1.3 *F. tularensis* Schu S4 Subverts the Innate Immune Response of the Host

**Figure 6:**
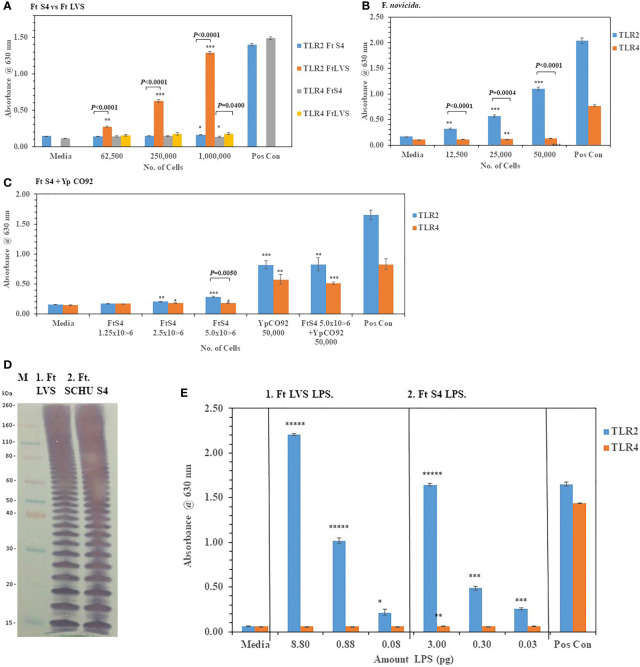
*F. tularensis* Schu S4 mitigates the activation of TLR2 and TLR4. Bacterial cell suspensions of *F. tularensis* (*Ft*) Schu S4, *Ft* LVS, and *F. novicida* were prepared from colonies in PBS after ~72 h of growth at 37°C on chocolate agar plates, and diluted bacterial cells were used to inoculate HEK293-TLR2 or -TLR4 cells. After 18−20 h of incubation, the amount of TLR2 and TLR4 activation was determined. **(A)** The activation of TLR2 or TLR4 by increasing amounts of *Ft* Schu S4 and *Ft* LVS was examined. **(B)** The activation of TLR2 or TLR4 by increasing amounts of *F. novicida* was examined. **(C)** The activation of TLR2 or TLR4 by increasing amounts of *Ft* Schu S4 was examined with and without *Y. pestis* (*Yp*) CO92 that was grown at 28°C on SBA plates for ~ 45 h. **(D)** Western blot (WB) analysis (10% Tricine gel) of LPS extracts from *Ft* Schu S4 (7 µl), lane 1, and *Ft* LVS (7 µl), lane 2. Note the numerous bands in both LPS preparations that may reflect the longer chain length of the O-antigen polysaccharide compared to other bacteria (see **Figure 5D**). **(E)** Activation of TLR2 and TLR4 by *Ft* Schu S4 (panel 1) or *Ft* LVS (panel 2) LPS extracts. All samples were analyzed in triplicate, and results represent one of two independent repeats. Results of TLR activation are presented as geometric mean with standard error of the mean. The media control was PBS, and positive controls (Pos Con) were HKLM (2 x 10^6^ cells) for TLR2, and *E. coli* LPS (0.2 ng) for TLR4. Significant differences in activation between cells are shown above the results. Significant values compared to media control are shown: *P ≤ 0.05; **P ≤ 0.01; ***P ≤ 0.001; ****P ≤ 0.0001; *****P ≤ 0.00001.

We then examined the ability of live *F. tularensis* (*Ft*) to activate TLR2 and TLR4 *Ft* Schu S4, *Ft* LVS, and *F*. *novicida* were grown on chocolate agar plates for three days at 37°C before adding different amounts of the bacteria to HEK293-TLR2 and -TLR4 cells. We saw significant activation of primarily TLR2 but not TLR4 compared to the media control cells by *Ft* LVS and *F. novicida* but not *Ft.* Schu S4 (**Figures 6A, B**). In a direct comparison between *Ft* Schu S4 and *Ft* LVS, we saw a significant difference (*P* < 0.0001) in TLR2 activation at all cell concentrations examined (**Figure 6A**). What little TLR4 activation by all three *Francisella* strains we saw was just above the media control cells even at the highest bacterial cell concentration used in the assay (50,000−1,000,000 cells). Because so little TLR2 or TLR4 activity by *Ft* Schu S4 was seen, we asked if the pathogen was antagonistic to the TLRs or the pathogen could interact with but did not activate TLRs. To answer these questions, we increased the number of *Ft* Schu S4 and co-incubated the *Ft Schu* S4 with another pathogen *Yp* CO92 (50,000 cells) grown at 28°C that we had previously shown to activate both TLR2 and TLR4 (see **Figure 2A**). Again, we saw very little activity of TLR2 and TLR4 with *Ft* Schu S4 (1.25−5.0 x 10^6^ cells), but there was significant activation of both TLR2 and TLR4 by *Yp* CO92 (50,000 cells) at the same time (**Figure 6C**). In addition, when both pathogens were incubated together, there was no noteworthy diminution of TLR2 and TLR4 activity compared to *Y*p CO92 by itself (**Figure 6C**). Because of the lack of activity by *Ft* Schu S4 cells we wanted to examine the LPS extract of the pathogen to see if there were differences with the LPS from *Ft* LVS, the live *Ft*. strain that activated TLR2. The banding pattern of the LPS from *Ft* LVS and *Ft* Schu S4 appeared to be similar (**Figure 6D**), and the qualitative activation of TLR2 but not TLR4 by the LPS extract from both organisms appeared to be similar (**Figure 6E**), although the LPS extracts from both organisms was assessed at different concentrations. We also noted that the number of bands was greater than what was observed with the *Bp* 1026b strain (see **Figure 5D**), which may reflect the increase in carbon chain length seen in Ft. strains compared to other bacteria (16 to 18 *vs.* 12 to 14, respectively) (Raetz et al., 2009; Okan and Kasper, 2013). Thus, the LPS extract from both stains stimulated TLR2 in a similar manner, but we saw no stimulation of TLR4 in either case. In summary, *Ft* Schu S4 apparently does not display any PAMPs to the host for surface assembled TLR2 or TLR4 (or TLR5, see below), when compared to the less pathogenic strains *Ft* LVS and *F*. *novicida*, which makes it a more subversive pathogen.

### 3.2 Activation of TLR5 by Live Gram-Negative Pathogens

#### 3.2.1 TLR5 Responds to Both Free Flagellin and Flagella Attached to *Burkholderia spp*

**Figure 7:**
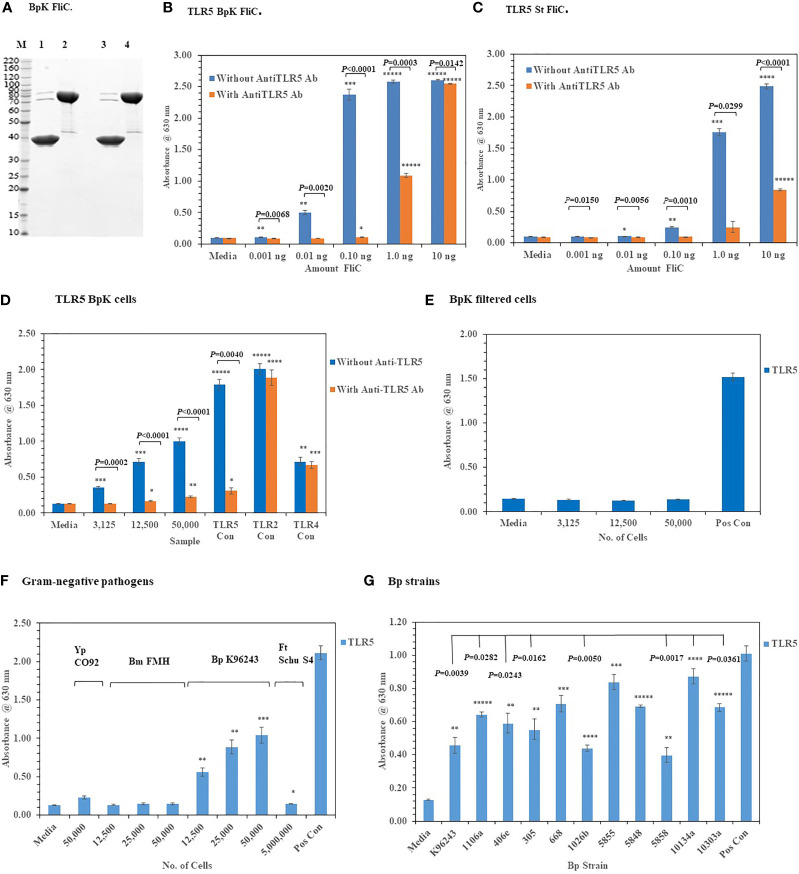
Activation of TLR5 by either flagellin or flagella. **(A)** Polyacrylamide gel electrophoresis (10.5−14% Criterion, Tris-HCl gel) of purified *Bp* K96243 flagellin preparations (5 µg each): M, marker; lane 1, FliC sample heated and reduced, prior to loading; lane 2, His6-MBP-tev-BpKFliC (uncleaved tev *Bp* K96243 FliC) sample heated and reduced, prior to loading; lane3, FliC, without heating or reducing; lane 4, His6-MBP-tev-BpKFliC without heating or reducing. **(B)** TLR5 activation by increasing amounts of *Bp* K96243 FliC with and without anti-TLR5 monoclonal antibody (100 ng). Significant differences in TLR5 activity with and without anti-TLR5 anti-TLR5 antibody are shown above. **(C)** TLR5 activation by increasing amounts of *St* FliC with and without anti-TLR5 monoclonal antibody (mAb) (100 ng). **(D)** Activation of TLR5 by live *Bp* K96243 cells can be inhibited by an anti-TLR5 mAb but not TLR2 or TLR4. Varying amounts of *Bp* K96243 cells was used to inoculate HEK293-TLR5 cells. Also, HEK 293 -TLR5, -TLR2, and -TLR4 cells with their respective positive control antigens were examined. All TLR activation samples were examined with and without anti-TLR5 mAb (100 ng). **(E)** Activation of TLR5 after filtration of *Bp* K96243 cells used in **Figure 7D**. Equivalent amounts of filtrate (in 20 µl) that contained 3,125, 12,500, and 50,000 *Bp* K96243 cells before filtration (0.2 µm) was added to HEK293-TLR5 cells and activation evaluated. The positive control was not filtered. **(F)** Activation of TLR5 by live Gram-negative pathogens. Gram-negative pathogens *Yp* CO92, *Bm* FMH, *Bp* K96243, and *Ft* Schu S4) were grown as described in the Material and Methods, and bacterial cell suspensions were used to inoculate HEK293-TLR5 cells. After overnight incubation (~20 h) at 37°C with 5% CO_2_, the absorbance of the plates were read at 630 nm. The media control was PBS, and the positive control (Pos Con) was *St* FliC (2 ng). **(G)** Activation of TLR5 by live cells of *Bp* spp. *Bp* spp. listed in **Table 1** were grown as described in the Material and Methods and used to inoculate HEK293-TLR5 cells as indicated. After overnight incubation (~20 h) at 37°C with 5% CO_2_, the absorbance of the plates were read at 630 nm. Although different amounts of cells were examined for TLR5 activity (6,250, 25,000, and 100,000 cells), for display purposes we only show the results for 100,000 cells, which gave the highest activity in all cases except one strain (Bp 406e). In this latter case, there was no statistical difference between the two highest cell concentration used. Statistical differences between the *Bp* strain with highest TLR5 activity (*Bp* HBPUB10134a) and the other strains is shown above in the figure. Results of TLR activation are presented as geometric mean with standard error of the mean. TLR5 activation samples were analyzed in triplicate and results represent one of two independent repeats. The media control was PBS. Significant differences between samples are shown above the results. Significant values compared to media control are shown: *P ≤ 0.05; **P ≤ 0.01; ***P ≤ 0.001; ****P ≤ 0.0001; *****P ≤ 0.00001.

We assessed the activation of TLR2 and TLR4 with live Gram-negative pathogens, we now wanted to examine the interaction of these pathogens with TLR5, which was another surface assembled TLR. TLR5 has been a well-studied PRR since it was first reported by Hayashi et al. (2001) that flagellin, which is the subunit of the assembled flagella, was the agonist for this TLR. To examine this interaction of live Gram-negative pathogens with HEK293-TLR5 cells, we first used a purified recombinant *Bp* K96243 (BpK) flagellin (~ 40 kDa) molecule (or FliC) to examine the interaction with HEK293-TLR5 cells (**Figure 7A**, lane 3). We examined 10-fold increasing concentrations of BpK FliC (0.001 ng to 1.0 ng) and saw significant activation of TLR5 beginning at 0.01 ng until it reached a maximum at 1.0 ng to 10.0 ng (*P* ≤ 0.01–0.00001) (**Figure 7B**). Activation of TLR5 could be largely inhibited by an anti-TLR5 monoclonal antibody (mAb)(100 ng) up to 1.0 ng of BpK FliC where almost 50% inhibition of activation was seen *(P* = 0.0003) but little inhibition at 10 ng of FliC (*P* = 0.0142). We saw a similar activation and inhibition of TLR5 with purified *S. typhimurium* (*St*) FliC, except it was not as dynamic as the BpK FliC (**Figure 7C**). Maximum activation of TLR5 by *St* FliC was not seen until at least 10 ng and just under half of TLR5 activation was inhibited at that FliC concentration with the anti-TLR5 mAb (*P* < 0.0001).

When we examined the activation of TLR5 with live, whole cells of BpK we saw a substantial increase in TLR5 activity with an increase of live, whole-cells of BpK (**Figure 7D**). To examine the specificity of live cells of BpK interacting with TLR5 more closely, we pre-incubated the HEK293-TLR5 cells with an anti-TLR5 mAb (100 ng) before adding increasing amounts of BpK cells and measured the activation of TLR5 after incubation (**Figure 7D**). We saw a significant decrease in TLR5 activation (*P* =0.0002 - < 0.0001) in all samples with live cells and the positive TLR5 control (*P* = 0.0040) compared to the same samples in the absence of the anti-TLR5 mAb. At the same time we saw no significant effects of the anti-TLR5 mAb on the activation of TLR2 or TLR4 control samples (**Figure 7D**). Because it had been previously proposed that only the flagellin molecule and not flagellin assembled into flagella can activate TLR5, we wanted to determine if TLR5 activation was associated (or attached) with the live bacterial cells. Thus, a portion of the BpK cells (shown in **Figure 7D**) was passed through a 0.2 um, low protein binding filter, and equivalent amounts of the filtrate that originally contained live bacterial cells was added to HEK293-TLR5 cells. We saw complete loss TLR5 activation in the cell-free filtrates (**Figure 7E**) suggesting that the source of TLR5 stimulatory activity (flagella) was associated with the bacterial cells.

After we validated the TLR5 activation of our HEK293-TLR5 cells, we examined the ability of other tier 1 Gram-negative pathogens of interest to activate TLR5. Of the four Gram-negative pathogens we examined (*Y. pestis* CO92, *B. mallei* FMH, *B. pseudomallei* K96243, and *Francisella tularensis* SCHU S4), only BpK showed significant activation of TLR5 as we had shown previously (**Figure 7F**). Although we saw that three out of the four Gram-negative pathogens did not activate TLR5, this was a further assessment of the activation of one of the three surface assembled TLRs. The one positive TLR5 result given by one of the pathogens was another confirmation of the accuracy of the cell culture system. We further evaluated 10 other *Bp* strains for their ability to activate TLR5. Although we saw varying amounts of activation of TLR5 by the *Bp* strains examined, all strains showed significant amounts of TLR5 activation compared to the media control in addition to BpK (**Figure 7G**). We show a representative example of the activation of TLR5 by 100,000 bacterial cells of each *Bp* strain (**Figure 7G**). The most active was *Bp* HBPUB10134a followed closely by *Bp* MSHR5855, while the least active was *Bp* MSHR5858. There was a statistical difference in the TLR5 activity between the most active strain and 7 out of 10 other *Bp* strains. There was no overall correlation between the amount of TLR5 activation and virulence of the *Bp* strain. For example, one of the most virulent *Bp* strains [*Bp* HBPUB10134a, LD_50_ ~1.0 CFU (Amemiya et al., 2015; Trevino et al., 2018)] showed the highest TLR5 activation level; however, the least virulent *Bp* strain (*Bp* 1106a, LD_50_ 4,270 CFU (Amemiya et al., 2015; Trevino et al., 2018) examined stimulated a moderate amount of TLR5 activity (*P* = 0.0282). *Bm* FMH did not activate TLR5 under the same conditions as the *Bp* strains or demonstrate motility on motility agar (data not shown), which confirmed the nonmotile nature of *Bm* FMH (Galyov et al., 2010).

#### 3.2.2 Deletion of *fli*C in Pathogenic BpK and Nonpathogenic *B. thailandensis* E264 Abolishes TLR5 Activation

**Figure 8:**
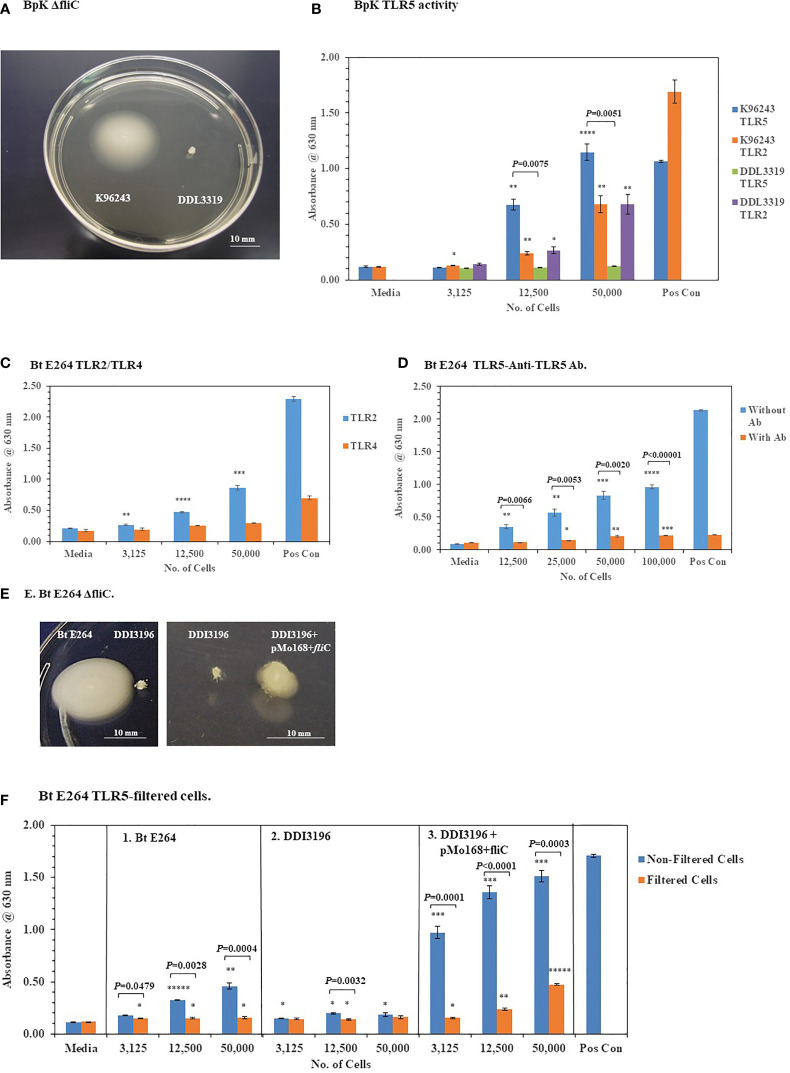
Deletion of *fli*C in pathogenic and nonpathogenic *Bp* strains abolishes TLR5 activation by the live microorganisms. **(A)** Wild-type *Bp* K96243 and *fli*C mutant DDI3319 on motility agar after 24 h. **(B)** Loss of the ability to activate TLR5 by the *fli*C mutant. Mutation in *fli*C does not affect activation of TLR2 by the *fli*C mutant. Significant differences in TLR5 activation by wild-type *Bp* K9624 and flicC mutant DDI3319 is shown above the activity. **(C)** Nonpathogenic *B. thailandensis* E264 activates primarily TLR2 similar to BpK. **(D)** Anti-TLR5 antibody significantly inhibits TLR5 activation by live *B. thailandensis* E264. **(E)** Mutation of *fli*C in *B. thailandensis* E264 (DDI3196) abrogates motility, but it can be partially restored (DDI3196+pMo168+*fli*C). **(F)** Filtration of *B. thailandensis* E264 abrogates TLR5 activation (panel 1). Filtration of cells and deletion of fliC in B. thailandensis E264 both abolishes TLR5 activation (panel 2). In the partially recombinant DDI3196+pMo168+*fli*C TLR5 activation is elevated, and TLR5 activity can be detected in the cell-free filtrate (panel 3). The media control was PBS, and positive controls (Pos Con) were *St* FliC (2 ng) for TLR5 and HKLM (2 x 10^6^ cells) for TLR2. The activation of TLRs was analyzed in triplicate and results represent one of two independent studies. Results of TLR activation are presented as geometric mean with standard error of the mean. Significant differences between samples are shown above the results. Significant values compared to media control are shown: *P ≤ 0.05; **P ≤ 0.01; ***P ≤ 0.001; *****P ≤ 0.00001.

To further validate the activation of TLR5 by BpK FliC the gene for flagellin was deleted, and the mutant isolated to evaluate it’s potential to activate TLR5. **Figure 8A** shows the phenotype of the wild-type BpK and *fli*C mutant (DDL3319) colonies after 24 h on motility agar. Motility by the *fli*C mutant was eliminated, and TLR5 activation was abolished when 12,500 and 50,000 *fli*C mutant cells were examined compared to BpK wild-type cells (*P* = 0.0075 and *P* = 0.0051, respectively) (**Figure 8B**). No *fli*C complemented mutant strain, however, could be isolated with the DDL3319 mutant. The activation of TLR2 was simultaneously measured by both wild-type and *fli*C mutant cells as a control to show that there was no difference between the strains.

To further substantiate that TLR5 activation could be associated with live bacterial cells, *fli*C was deleted in *B. thailandensis* (*Bt*) E264, which is a motile, nonpathogenic, environmental microorganism (Brett et al., 1998). Similar to *Bp cells Bt* E264 cells activated primarily TLR2 but little TLR4 (**Figure 8C**). In addition, we saw a significant inhibition of TLR5 activation by *Bt* E264 cells (*P* = 0.0066 − ≤ 0.00001) in the presence of anti-TLR5 antibody compared to the amount of activation without the anti-TLR5 antibody (**Figure 8D**). When *fli*C was deleted in *Bt* E264 we saw a loss of motility by the mutant (DDI3196) compared to the wild-type strain (**Figure 8E**) which confirmed the mutation in *fli*C. In a partial *fli*C complemented strain of *Bt* E264 (DDI3196+pMo168+*fli*C) there was partial restoration of motility compared to the *fli*C mutant DDI3196 (**Figure 8E**).

To assess the association of TLR5 activation with *Bt* E264 cells, we took bacterial cell suspensions of the wild-type *Bt* E264, of the *fli*C mutant DDI3196, and of the partial *fli*C complemented strain DDI3196 (pMo168+*fli*C), and passed each through a 0.2 um filter. Equivalent amounts of filtrate that previously contained bacterial cells were examined for their ability to activate TLR5 compared with unfiltered cells. Filtration of increasing amounts of wild-type Bt E264 cells abrogated TLR5 activation (*P* = 0.0479 – 0.0004) (**Figure 8F**, panel 1). Mutation of *fli*C also eliminated TLR5 activation by the DDI3196 mutant similar to elimination of bacterial cells as filtration (**Figure 8F**, panel 2). In the partially complemented *fli*C mutant (DDI3196+pMo168+*fli*C), we saw a substantial increase in TLR5 activation over that seen in the wild-type cells at all cell concentrations examined in the unfiltered samples (**Figure 8F**, panel 1 *vs* panel 3). Furthermore, there was a notable increase in TLR5 activation by the cell-free filtrate compared to the unfiltered samples, especially with the filtrate that originally contained 12,500 (*P* < 0.0001) and 50,000 (*P* = 0.0003) cells (**Figure 8F**, panel 3). The latter result suggests the possibility that not all flagellin produced by the pMo168+*fli*C expression plasmid was assembled into flagella (which partly restored flagella biosynthesis), and excess flagellin may be secreted or leaked by the partially complemented *fli*C cells and can be found in the cell-free filtrate.

#### 3.2.3 *B. pseudomallei* K96243 and *B. thailandensis* E264 FliC Share a Critical Region With *S. typhimurium* LT2 FliC Required for Interaction With TLR5

**Figure 9:**
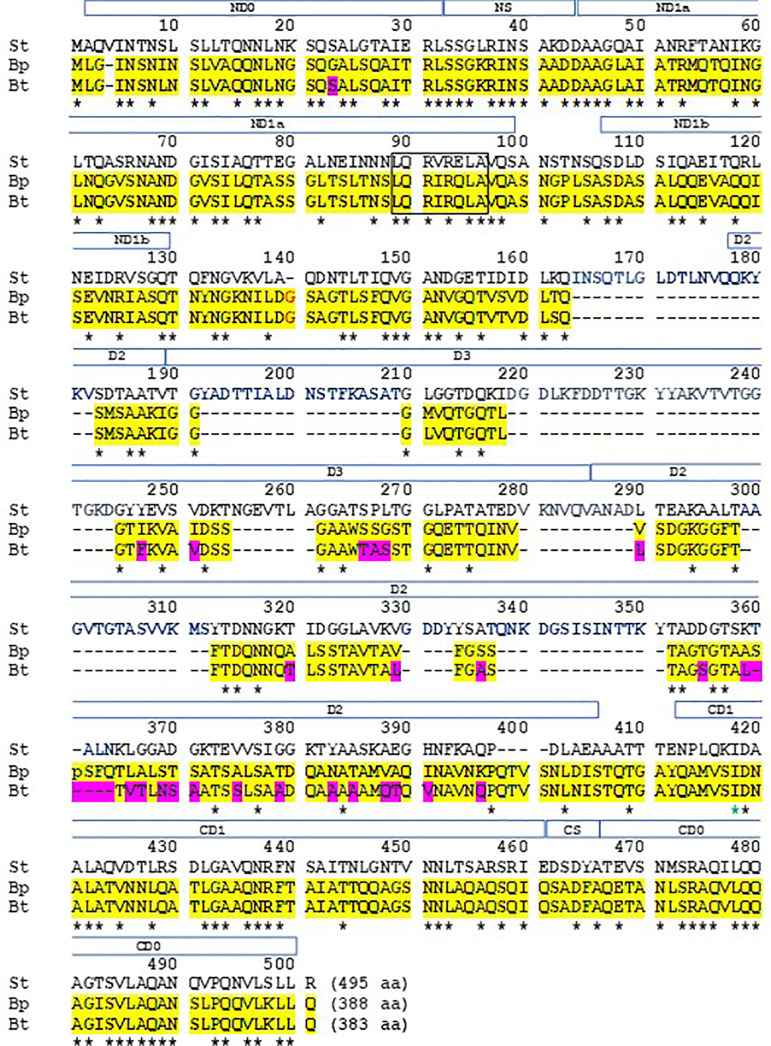
Alignment of amino acid sequences of *St* LT2, *Bp* K96243, and *Bt* E264 FliC reveals common sequences required for folding and TLR5 activation. The amino acid sequences of FliC (495 aa, 388 aa, and 383 aa, respectively) from the three microorganisms were aligned for optimal identity (Papadopoulos and Agarwala, 2007). The amino acid sequences of FliC of *Bp* K96243 and *Bt* E264 are highlighted in yellow with differences between the two sequences highlighted in red. The asterisk below the three sequences represent identical amino acids for all three FliCs. The dashes are spaces between amino acids for missing amino acids in the three FliCs. The amino acids within the square (89−96 aa) have been reported to be required for recognition by TLR5 (Smith et al., 2003; Andersen-Nissen et al., 2005). The open boxed region above the sequences represent structural domains (D0, D1, D2, and D3) of FliC that are involved in tertiary formation of the molecule and are found in both N- and C-terminal regions (regions preceded by N or C, respectively) of the molecule. (Smith et al., 2003; Yonekura et al., 2003; Andersen-Nissen et al., 2005; Yoon et al., 2012). Within the domains there are subregions, and between D0 and D1 there is a small “spoke” (S) region at both N-terminal (NS) and C-terminal regions (CS) (Smith et al., 2003; Yonekura et al., 2003; Andersen-Nissen et al., 2007; Yoon et al., 2012).

We wanted to examine the primary amino acid sequence of FliC in BpK and *Bt* E264 for the presence of the amino acid sequence that appears to be required for TLR5 activation by *St* (Smith et al., 2003; Andersen-Nissen et al., 2005). The amino acid sequence of BpK and *Bt* E264 FliC are 388 and 383 in length, respectively, whist *St* LT2 FliC was 495 (**Figure 9**). Between BpK and *Bt* E264 FliC, there was 93% identity with most of the differences was found near the C-terminal region of FliC. *St* LT2 FliC had been divided into 4 domains, D0, D1, D2, and D3, which are designated above the amino acid sequence (open bar) shown in **Figure 9**. Domains D0 and D1 of *St* LT2 FliC are located at the N- and C-terminal regions of the protein (ND0, ND1, and CD0, CD1, respectively). In contrast, BpK and *Bt* E264 FliC are missing 112 amino acids present in the *St* LT2 FliC regions D2 and D3. In *Burkholderia* FliC, we found 10/12 identical amino acids in the NS but only 2/5 identical amino acids in the CS region. The first 163 amino acids of the *Burkholderia* FliCs was 51.5% identical with *St* LT2 FliC, and the last 83 amino acids of the *Burkholderia* FliCs was 56.6% identical with *St* LT2 FliC. In addition, within ND1 of *St* FliC amino acids 89 – 96, was the essential sequence that was reported to interact with TLR5 (Smith et al., 2003; Andersen-Nissen et al., 2005). In both *Burkholderia* FliCs there was 6/8 identical amino acids in the same region. Thus, although there was a disparity in the size of FliC between the microorganisms, they shared common regions needed for tertiary formation of FliC and assembly of the flagellum, and they shared a critical primary sequence that was required for TLR5 activation.

## 4 Discussion

Our aim was to assess how live, Tier 1 Gram-negative bacterial cells interacted with cell surface expressed TLRs, which we believe to be representative of the early encounter of the pathogen with the host. In our initial study we investigated the hypothesis that *Yp* can alter the biosynthesis of lipid A depending on growth at 28 C (more reactive lipid A) or 37 C (less reactive lipid A). We could directly detect the changes in TLR2 or TLR4 activation with whole cells of *Yp* at the two different temperatures that likely reflected the changes in lipid A biosynthesis. The changes in lipid A biosynthesis at these two temperatures have been well documented with hexa-acyl lipid A being synthesized at 28 C (Kawahara et al., 2002; Rebeil et al., 2004; Montminy et al., 2006; Matsuura et al., 2010). At the higher temperature of 37°C associated with mammalian infection, TLR2 was primarily activated with concurrent suppression of TLR4 activation that coincided with the biosynthesis of tetra- or penta-acylated modified forms of lipid A as previously reported (Kawahara et al., 2002; Rebeil et al., 2004; Montminy et al., 2006; Matsuura et al., 2010). We were not able to observe the same temperature effects on TLR2 or TLR4 activation by *Bp* K96243 or *Bm* FMH. This observation supports a recent report that growth of *Bp* K96243 at different cultural conditions did not alter lipid A modification and that by MALDI-TOF lipid A spectra analysis tetra- and penta-acylated species were consistently present (Sengyee et al., 2018).

Another observation we noted using live, whole cells was the mitigation of TLR2 and TLR4 activation by the *Bp* 1026b capsule. The capsule of *Bp* and the closely related *Bm* is a virulence factor and contributes to resistance to serum killing, decrease in opsonization, and persistence in infected hosts (DeShazer et al., 2001; Atkins et al., 2002; Reckseidler-Zenteno et al., 2005; Warawa et al., 2009; Zhang et al., 2011; Heiss et al., 2012; Woodman et al., 2012). These anti-microbial properties of the capsule can be attributed in part to the prevention of complement C3 and antibody deposition on the pathogen (Reckseidler-Zenteno et al., 2005; Zhang et al., 2011; Woodman et al., 2012). We now showed that capsule can attenuate the early innate immune response of the host to the presence of the pathogen. This in turn could directly affect the expression of cytokines/chemokines needed for the early recruitment of innate immune cells, such as phagocytic macrophages and neutrophils, and NK cells, to limit the spread of the invading pathogen (Woodman et al., 2012; Wiersinga et al., 2018). Thus, we have revealed another important immune property of the capsule that contributes to the virulence of these Gram-negative pathogens.

Our results, however, were different from a previous report that found no significant difference in TLR4 activation by a capsule negative *Bp* compared to the wild-type *Bp* strain (Sengyee et al., 2018). One major difference between our study and that of Sengyee et al. (2018) was that they used heat-inactivated bacterial cells (80°C for 1 h) to assess TLR4 activation, while we used live *Bp* in our studies. High temperatures have been reported to cause changes in the outer membrane and loss of LPS of Gram-negative bacteria (Russell, 2003). In addition, heating (70°C for 15 min) depolymerized flagella of *Salmonella typhimurium* which would alter its interaction with TLR5 (Smith et al., 2003). We did see another difference in our studies with *Bp* 1026b wild-type and capsule mutant LPS extract in the activation of TLR2 or TLR4 as previously observed (West et al., 2008). They reported that their *Bp* LPS preparations activated TLR4 and not TLR2, and that TLR4 activation was dependent on CD14 and MD-2. In our studies we saw a higher TLR2 response than a TLR4 response to LPS from both Bp 1026b wild-type and capsule mutant LPS extracts, which was similar to the TLR response to live *Bp* cells. On closer examination of their report it appears that their *Bp* LPS (and lipid A) preparations were more highly purified than the *Bp* LPS extracts used in our studies. They eliminated the presence of phospholipids and other lipoproteins that could activate TLR2. Nevertheless, we did not see any apparent differences between the wild-type and capsule mutant LPS extracts by gel analysis and TLR activity.

Our studies with *Francisella* strains revealed the absence of TLR4 activation compared to the other Gram-negative pathogens in the current study. This was most striking with *Ft* Schu S4 (a type A strain) cells where even TLR2 activation was absent. *Ft* Schu S4 had been previously reported to show little stimulation of the innate immune response in human dendritic cell culture (Chase et al., 2009), or in a microarray analysis of infected human monocytes (Butchar et al., 2008). The lack of TLR2 and TLR4 activation by *Ft* Schu S4 cells may reflect in part the presence of an unusual lipid A moiety associated with *Francisella* spp. (Wang X. et al., 2006; Raetz et al., 2009). Approximately 90% of lipid A embedded in the outer membrane has been described as “free lipid A” which was present in *Ft* LVS and *F*. *novicida* (Wang X. et al., 2006). This fraction of lipid A lacks Kdo and hence is not glycosylated with core subunits, or contains O-antigen sugars. Another major difference in lipid A in *Francisella* is the presence of only tetraacylated chains that are 16 and 18 carbons in length compared to the variable number of acylated chains that are typically 12 to 14 carbons in length in many bacteria (Raetz et al., 2009; Okan and Kasper, 2013). In contrast to *Ft* Schu S4, *Ft* LVS (a type B strain) and *F. novicida*, which are not considered human pathogens, exhibited significant TLR2 activation. LPS prepared from *Ft* Schu S4 and *Ft* LVS, also activated TLR2. Because *Ft* Schu S4 cells were negative for activation of TLR2 and TLR4, it is likely that TLR2 activation by LPS from *Ft* Schu S4 is from the presence of tri-acylated lipoproteins, or other TLR2 activating polysaccharides, for example capsule, in the LPS extract (Kim et al., 1996; Cole et al., 2006; Wang et al., 2006; Cole et al., 2007; Butchar et al., 2008; Thakran et al., 2008). We also do not know how these components are organized on the surface of *Ft* Schu S4 and *Ft* LVS, however, because we see differences between the activation of TLR2 by the two different Ft species, it suggests that organization of the outer surface of the microorganisms is different. Tri-acylated lipoproteins have been generally associated with the outer membranes of Gram-negative bacterial and activate the heterodimer TLR1/TLR2, although some Gram-positive microorganisms are able to synthesize both di-and tri-acylated lipoproteins (Takeuchi et al., 2002; Jin et al., 2007; Thakran et al., 2008; Kovacs-Simon et al., 2011; Nakayama et al., 2012). On the other hand, di-acylated lipoproteins are recognized only by the TLR1/TLR6 heterodimer. LPS from *Ft* LVS and *F. novicida* had previously been reported to show no activation of TLR2 or TLR4 (Cole et al., 2006; Hajjar et al., 2006), therefore, activation of TLR2 with cells could be from the lipoproteins or other glycoproteins associated with the outer membrane of the cell (Thakran et al., 2008). Since *Ft* Schu S4 cells did not activate TLR2 or TLR4 it implicates the presence of another component, such as a capsule or envelope that shields the pathogen from the host’s innate immune response differently from that in the less pathogenic *Francisella* strains (*Ft* LVS or *F. novicida*) (Rowe and Huntley, 2015).

Our observation using live, whole cells with flagella attached to *Bp* K96243 or *Bt* E264 activated TLR5 was noteworthy. Activation of TLR5 was authenticated in a number of ways: 1. Purified FliC from *St* or *Bp* K96243 activated TLR5; 2. An anti-TLR5 mAb inhibited TLR5 activation by FliC from both these microorganisms but did not affect TLR2 or TLR4 activation; 3. The anti-TLR5 mAb also eliminated TLR5 activation by whole-cells of motile pathogenic *Bp* K96243 and motile nonpathogenic *Bt* E264; 3. Filtration of *Bp* K96243 and *Bt* E264 cells through a 0.2 um filter eliminated TLR5 activation; and 4. Deletion of *fli*C in both *Burkholderia* species abrogated TLR5 activation by both mutants. A high resolution crystal structure of *Bp* K96243 FliC (residues 25 to 378) has been reported (Nithichanon et al., 2015). The modeled FliC structure contained the folded-domains consisting of the N- and C-terminal regions that are adjacent to each other as found in the *St* FliC crystal structure. It was not clear if the consensus sequence required for TLR5 activation would be available in this FliC model.

Activation of TLR5 by *Bp* 1026b FliC has been previously reported (Birnie et al., 2019; Dickey et al., 2019). Whether activation of TLR5 by flagella can occur, or even the possibility of flagella attached to bacterial cells can activate TLR5 has been in doubt (Smith et al., 2003; Andersen-Nissen et al., 2005). Smith et al. (2003) showed that TLR5 activation by filamentous flagellin (flagella) was much less compared to flagellin; however, it still could activate TLR5 albeit at a lower activity. On a weight basis flagellin activated TLR5 3-5 fold more than flagella filaments (~60-fold *vs* 20-fold activation at ~1 ng/ml, respectively). It was also suggested that heating (70°C, 15 min) flagella filaments disassembled the structure and released flagellin subunits. It was not clear to what extent disassembly would occur. In a follow up study heat-inactivated, flagellated bacteria was used to examine TLR5 activation by bacteria from different classifications (Andersen-Nissen et al., 2005).

Our results suggest that either *Burkholderia* flagellin or flagella can activate TLR5. For activation of TLR5 to occur the flagellin molecule must occupy a binding site on each TLR5 monomer prior to homodimer formation and eventual activation of MyD88-dependent signaling to activate NF-κB (Yoon et al., 2012; Ivicak-Kocjan et al., 2013). Consequently, for TLR5 dimer formation to occur with a flagellum it may take one (linking both TLR5 monomers) or two flagellums with each occupying a FliC binding domain on a TLR5 monomer to initiate the dimerization and activation process. These proposed events with flagella would be a more complex process than that initiated with flagellin molecules. This could explain the difference in the ability of soluble flagellin compared to insoluble flagella to activate TLR5 (Smith et al., 2003). Also, because the sequence of *Bp* K96243 FliC was not identical to that of *St* FliC (33% identical overall), the TLR5 recognition site in *Bp* K96243 FliC may be more accessible than in *St* FliC. We did see that the *Bp* K96243 FliC could activate TLR5 at a higher rate than an equivalent amount of *St* FliC (see **Figure 7**), even after considering the differences in mass of the FliC molecules. More studies will be needed to further confirm our observation of activation of TLR5 by the *Bp* flagellum or by other strains. In contrast, there are motile pathogenic bacterial strains that have evolved to avoid activating the host’s TLR5, such as *Helicobacter pylori* and *Campylobacter jejuni*, that have changes in the critical 89 – 96 amino acid region of FliC needed for TLR5 activation (Ramos et al., 2004; Andersen-Nissen et al., 2005). It would be an advantage for the host to have the ability to detect either flagellin or flagellum to mitigate infection by a motile pathogen. Heating to inactivate the pathogen may well raise questions of the quality of the interaction with TLRs. Nevertheless, microorganisms have been heat-inactivated to examine their interaction with TLR 5 (or other TLRs) (Andersen-Nissen et al., 2005; West et al., 2008; West et al., 2013; Chaichana et al., 2017; Birnie et al., 2019; Dickey et al., 2019). Hence, we used two methods in the present study to examine intact flagella interaction with TLR5. Firstly, we used live, motile *Bp* cells to inoculate HEK293-TLR5 cells, and secondly, a filter (0.2 µm) to exclude motile bacteria (with flagella attached, see **Figure 1**) to demonstrate that TLR5 activation could be abolished. This was confirmed with two different species of *Burkholderias*. It cannot be ruled out that there was some free flagellin in our cell suspension, however. One exception where eliminating bacterial cells by filtration did not completely abolish TLR5 activation in the cell-free filtrate was in the study with the *Bt* E264 (DDI3196+pMo168+*fli*C) partially complemented *fli*C mutant (**Figure 8F**). With the complemented *fli*C mutant, we saw a large increase in TLR5 activation compared to that of the wild-type parent strain. After a suspension of the complemented *fli*C mutant was passed through a 0.2 um filter, TLR5 activation was abrogated, except at the higher cell concentrations where we saw an increase in TLR5 activity in an equivalent amount of filtrate. We believe this to be FliC present in the filtrate that was over produced by the expression plasmid pMo168+*fli*C and, subsequently, secreted or leaked from the complemented mutant. Further studies are needed to examine the phenotype of the complemented *fli*C strain more closely as regards to the presence of flagella and to examine the efficacy of filtration of other motile microorganisms to prevent TLR5 activation.

Although in the past many interactions of TLRs have been evaluated with purified agonist in the absence of the microorganism, our results suggest that early activation of TLRs may also occur when PAMPs are still associated with a live infectious agent. We believe this to be a possibility in the initial stages of infection even before the invading agent has established an active infection. It has been suggested that the agonist must be dissociated (or secreted) from the microorganism, for example during growth, before it can interact with TLRs (Tesh et al., 1986; Zhang et al., 1998; Hellman et al., 2000; Kitchens et al., 2000; Vesy et al., 2000; Matsuura, 2013). Possibly, both free and associated PAMPs can activate TLRs during the early period of infection of the host by the pathogen. On the other hand, the agonist might dissociate from the microorganism after binding to the TLR or with the participation of an unidentified enzyme/mechanism that can free the agonist from the surface of the microorganism. Without the free agonist, the TLR4 endocytic pathway (TRIF-dependent) would be excluded. Further studies are needed, for example cross-linking studies, to examine the early interaction of the microorganism with TLRs to resolve some of the possible mechanisms for TLR activation in the host.

In conclusion, it has become more apparent in the evolution of successful pathogenic bacteria that avoidance of the host’s TLR4 response is a common theme (Arpaia and Barton, 2013). Taken together the host’s surface expressed TLRs, TLR2 (TLR1/2, TLR2/6), TLR4, and TLR5, all recruit a common cytosolic signal transduction protein MyD88 that leads to the eventual activation of NF-κB and AP-1. Therefore, why have pathogens appear to commonly evolved mechanisms to suppress or avoid activation of TLR4? It may be because activation of TLR4 can lead to two host innate immune responses to the pathogen: a MyD88-dependent response and a TRIF-dependent response. The alternative TLR4 endocytic pathway or TRIF-dependent response leads to the production of anti-microbial type 1 interferons (IFNα/β) and delayed activation of NF-κB (Barton and Medzhitov, 2003; Kagan et al., 2008). Depending on the pathogen, IFNα/β appears to restrict pathogen growth, activate macrophages, and upregulate protective cytokines, although there were some reports of detrimental effects on the host (McNab et al., 2015). Studies on the early interaction of live pathogenic microorganisms with specific TLRs could augment the discovery of new therapeutic compounds for the treatment of infectious diseases.

## Data Availability Statement

The original contributions presented in the study are included in the article/**Supplementary Material**. Further inquiries can be directed to the corresponding authors.

## Author Contributions

KA and DD designed, performed experiments, and wrote the manuscript. RB and JD performed experiments and contributed to writing the manuscript. DF performed the statistical analyses of the studies and contributed to the manuscript. DW was involved in the design of the studies and contributed to the manuscript. PW was involved in the design, funding, administration of the studies and contributed to the manuscript. All authors approved the submitted version of the manuscript.

## Funding

The authors would like to thank JSTO/Defense Threat Reduction Agency for their support of USAMRIID project no. 923678.

## Conflict of Interest

The authors declare that the research was conducted in the absence of any commercial or financial relationships that could be construed as a potential conflict of interest.

## Publisher’s Note

All claims expressed in this article are solely those of the authors and do not necessarily represent those of their affiliated organizations, or those of the publisher, the editors and the reviewers. Any product that may be evaluated in this article, or claim that may be made by its manufacturer, is not guaranteed or endorsed by the publisher.

## Supplementary Material

Supplementary Figure 1 HEK293-TLR2 and HEK293-TLR4 cells show high specificity to their specific agonist. HEK TLR2 and HEK TLR4 cells were grown and harvested as described in the Methods section. The cells were inoculated into 96-well plates in triplicate and incubated with their respective agonist, HKLM (2 x 10^6^ cells) for HEK TLR2 cells or Ec LPS (0.2 ng) for HEK TLR4 cells. Another set of TLR2 cells were incubated with Ec LPS and another set of TLR4 cells were incubated with HKLM. After overnight incubation at 37°C with 5% CO_2_ the plates were read at 630 nm. Significant differences between the agonist on the specific cell line are shown above the data. Results of TLR activation are presented as geometric mean with standard error of the mean. Significant values compared to media control are shown: ****P* ≤ 0.001; *****P* ≤ 0.0001.

## References

[B1] Akira S. Uematsu S. Takeuchi O. (2006). Pathogen Recognition and Innate Immunity. Cell 124, 783–801. doi: 10.1016/j.cell.2006.02.015 16497588

[B2] Amemiya K. Dankmeyer J. L. Fetterer D. P. Worsham P. L. Welkos S. L. Cote C. K. (2015). Comparison of the Early Host Immune Response to Two Widely Diverse Virulent Strains of Burkholderia Pseudomallei That Cause Acute or Chronic Infections in BALB/c Mice. Microbial Path. 86, 53–63. doi: 10.1016/j.micpath.2015.07.004 26162294

[B3] Andersen-Nissen E. Smith K. D. Strobe K. L. Barrett S. L. R. Cookson B. T. Logan S. M.. (2005). Evasion of Toll-Like Receptor 5 by Flagellated Bacteria. Proc. Natl. Acad. Sci. 102, 9247–9252. doi: 10.1073/pnas.0502040102 15956202 PMC1166605

[B4] Andersen-Nissen E. Smith K. D. Bonneau R. Strong R.K. Aderem A. (2007). A Conserved Surface on Toll-Like Receptor 5 Recognizes Bacterial Flagellin. J. Exp. Med. 204, 393–403. doi: 10.1084/jem.20061400 17283206 PMC2118731

[B5] Arpaia N. Barton G. M. (2013). The Impact of Toll-Like Receptors on Bacterial Virulence Strategies. Curr. Opin. Microbiol. 16, 17–22. doi: 10.1016/j.mib.2012.11.004 23290772 PMC3622189

[B6] Asmar A. T. Collet J-F. (2018). Lpp, the Braun Lipoprotein, Turns 50 – Major Achievements and Remaining Issues. FEMS Microbiol Lett. 365, fny199. doi: 10.1093/female/fny199 30107563

[B7] Atkins T. Prior R. Mack K. Russell P. Nelson M. Prior J.. (2002). Characterization of an Acapsular Mutant of *Burkholderia pseudomallei* Identified by Signature Tagged Mutagenesis. J. Mol. Microbiol. 51, 539–547. doi: 10.1099/0022-1317-51-7-539 12132769

[B8] Barton G. M. Medzhitov R. (2003). Toll-Like Receptor Signaling Pathways. Science 300, 1524–1525. doi: 10.1126/science.1085536 12791976

[B9] Beatson S. A. Minamino T. Pallen M. J. (2006). Variation in Bacterial Flagellins: From Sequence to Structure. Trend. Microbiol. 14, 151–155. doi: 10.1016/j.tim.2006.02.008 16540320

[B10] Birnie E. Weehuizen T. A. F. Lankelma J. M. de Jong H. K. Koh G. C. K. W. van Lieshout M. H. P.. (2019). Role of Toll-Like Receptor 5 (TLR5) in Experimental Melioidosis. Infect. Immun. 87, e00409–e00418. doi: 10.1129/IAI.00409-18 31109950 PMC6652761

[B11] Braun V. Rehn K. (1969). Chemical Characterization, Spatial Distribution and Function of a Lipoprotein (Murein-Lipoprotein) of the *E. coli* Cell Wall. The Specific Effect of Trypsin on the Membrane Structure. Eur. J. Biochem. 10, 426–438. doi: 10.1111/j.1432-1033.1969.tb00707x 4899922

[B12] Brett P. J. DeShazer D. Woods D. E. (1998). *Burkholderia thailandensis* Sp. Nov., A *Burkholderia Pseudomallei*-Like Species. Internat. J. System. Bacteriol. 48, 317–320. doi: 10.1099/00207713-48-1-317 9542103

[B13] Burtnick M. Brett P. J. Harding S. V. Ngugi S. A. Ribot W. J. Chantratita N.. (2011). The Cluster 1 Type VI Secretion System Is a Major Virulence Determinant in *Burkholderia pseudomallei* . Infect. Immun. 79, 1512–1525. doi: 10.1128/IAI.01218-10 21300775 PMC3067527

[B14] Butchar J. P. Cremer T. J. Clay C. D. Gavrilin M. A. Wewers M. D. Marsh C. B.. (2008). Microarray Analysis of Human Monocytes Infected With *Francisella tularensis* Identifies New Targets of Host Response Subversion. PloS One 3 (8), e2924. doi: 10.1371/journal.pone.0002924 18698339 PMC2488368

[B15] Chaichana P. Chantratita N. Brod F. Koosakulnirand S. Jenjaroen K. Chumseng S.. (2017). A Nonsense Mutation in TLR5 Is Associated With Survival and Reduced IL-10 and TNF-α Levels in Human Melioidosis. PloS Negl. Trop. Dis. 11 (5), e0005587. doi: 10.1371/journal.pntd.0005587 28475641 PMC5435357

[B16] Chase J. C. Celli J. Bosio C. M. (2009). Direct and Indirect Impairment of Human Dendritic Cell Function by Virulent *Francisella tularensis* Schu S4. Infect. Immun. 77, 180–195. doi: 10.1128/IAI.00879-08 18981246 PMC2612294

[B17] Cheng A. C. Currie B. J. (2005). Melioidosis: Epidemiology, Pathophysiology, and Management. Clin. Microbiol. Rev. 18, 383–416. doi: 10.1128/CMR.18.2.383-416.2005 15831829 PMC1082802

[B18] Cole L. E. Elkins K. L. Michalek S. M. Oureshi N. Eaton L. J. Rallabhandi P.. (2006). Immunologic Consequences of *Francisella tularensis* Live Vaccine Strain Infection: Role of the Innate Immune Response in Infection and Immunity. J. Immunol. 176, 6888–6899. doi: 10.4049/jimmunol.176.11.6888 16709849

[B19] Cole L. E. Shirey K. A. Barry E. Santiago A. Rallabhandi P. Elkins K. L.. (2007). Toll-Like Receptor 2-Mediated Signaling Requirements for *Francisella tularensis* Live Vaccine Strain Infection of Murine Macrophages. Infect. Immun. 75, 4127–4137. doi: 10.1128/IAI.01868-06 17517865 PMC1951974

[B20] Cowles C. E. Yongfeng L. Semmelhack M. F. Cristea I. M. Silhavy T. J. (2011). The Free and Bound Forms of Lpp Occupy Distinct Subcellular Locations in *Escherichia coli* . Mol. Microbiol. 79, 1168–1181. doi: 10.1111/j.1365-2958.2011.07539.x 21219470 PMC3090202

[B21] DeShazer D. Waag D. M. Fritz D. L. Woods D. E. (2001). Identification of a *Burkholderia Mallei* Polysaccharide Gene Cluster by Subtractive Hybridization and Demonstration That the Encoded Capsule Is an Essential Virulence Determinant. Microbial Pathog. 30, 253–269. doi: 10.1006/mpat.2000.0430 11373120

[B22] Dickey A. K. Chantratita N. Tandhavanant S. Ducken D. Lovelace-Macon L. Seal S.. (2019). Flagellin-Independent Effects of a Toll-Like Receptor 5 Polymorphism in the Inflammatory Response to Burkholderia Pseudomallei. PloS Negl. Trop. Dis. 13 (5), e0007354. doi: 10.1371/journal.pntd.0007354 31067234 PMC6527242

[B23] Donnelly M. A. Steiner T. S. (2002). Two Nonadjacent Regions in Enteroaggregative *Escherichia coli* Flagellin Are Required for Activation of Toll-Like Receptor 5. J. Biol. Chem. 277, 40,456–40,461. doi: 10.1074/jbc.M206851200 12185085

[B24] Duan Q. Zhou M. Zhu L. Zhu G. (2013). Flagella and Bacterial Pathogenicity. J. Basic Microbiol. 53, 1–8. doi: 10.1002/jobm.201100335 22359233

[B25] Ellis J. Oyston P. C. F. Green M. Titball R. W. (2002). Tularemia. Clin. Microbiol. Rev. 15, 631–646. doi: 10.1128/CMR.15.4.631-646.2002 12364373 PMC126859

[B26] Forstneric V. Ivicak-Kocjan K. Ljubetic A. Jerala R. Bencina M. (2016). Distinctive Recognition of Flagellin by Human and Mouse Toll-Like Receptor 5. PloS One 11 (7), e0158894. doi: 10.1371/journal.pone.0158894 27391968 PMC4938411

[B27] Galyov E. E. Brett P. J. DeShazer D. (2010). Molecular Insights Into Burkholderia Pseudomallei and *Burkholderia mallei* Pathogenesis. Annu. Rev. Microbiol. 64, 495–517. doi: 10.1146/annurev.micro.112408.134030 20528691

[B28] Haiko J. Westerlund-Wikstrom B. (2013). The Role of the Bacterial Flagellum in Adhesion and Virulence. Biology 2, 1242–1267. doi: 10.3390/biology/2041242 24833223 PMC4009794

[B29] Hajjar A. M. Harvey M. D. Shaffer S. A. Goodlett D. R. Sjostedt A. Edebro H.. (2006). Lack of *In Vitro* and *In Vivo* Recognition of *Francisella tularensis* Subspecies Lipopolysaccharide by Toll-Like Receptors. Infect. Immun. 74, 6730–6738. doi: 10.1128/IAI.00934-06 16982824 PMC1698081

[B30] Hamad M. A. Zajdowicz S. L. Holmes R. K. Voskuil M. (2009). An Allelic Exchange System for Compliant Genetic Manipulation of the Select Agents *Burkholderia pseudomallei* and *Burkholderia mallei* . Gene 430, 123–131. doi: 10.1016/j.gene.2008.10.011 19010402 PMC2646673

[B31] Hayashi F. Smith K. D. Ozinsky A. Hawn T. R. Yi E. C. Goodlett D. R.. (2001). The Innate Immune Response to Bacterial Flagellin Is Mediated by Toll-Like Receptor 5. Nature 410, 1099–1103. doi: 10.1038/35074106 11323673

[B32] Heiss C. Burtnick M. N. Wang Z. Azadi P. Brett P. J. (2012). Structural Analysis of Capsular Polysaccharide Expressed by *Burkholderia mallei* and *Burkholderia pseudomallei* . Carbohyd. Res. 349, 90–94. doi: 10.1016/j.carres.2011.12.011 22221792

[B33] Hellman J. Loiselle P. M. Tehan M. M. Allaire J. E. Boyle L. A. Kurnick J. T.. (2000). Outer Membrane Protein A, Peptidoglycan-Associated Lipoprotein, and Murein Lipoprotein Are Released by *Escherichia coli* Bacteria Into Serum. Infect. Immun. 68, 2566–2572. doi: 10.1128/IAI.68.5.2566-2572.2000 10768945 PMC97460

[B34] Hinnebusch B. J. Jarrett C. O. Bland D. M. (2017). “Fleaing” the Plague: Adaptations of *Yersinia pestis* to Its Insect Vector That Lead to Transmission. Annu. Rev. Microbiol. 71, 215–232. doi: 10.1146/annurev-micro-090816-093521 28886687

[B35] Inouye M. Shaw J. Shen C. (1972). The Assembly of a Structural Lipoprotein in the Envelope of Escherichia Coli. J. Biol. Chem. 247, 8154–8159. doi: 10.1016/S0021-9258(20)81822-5 4565677

[B36] Ivicak-Kocjan K. Panter G. Bencina M. Jerala R. (2013). Determination of the Physiological 2:2 TLR5:flagellin Activation Stoichiometry Revealed by the Activity of a Fusion Receptor. Biochem. Biophys. Res. Commun. 435, 40–45. doi: 10.1016/j.bbrc.2013.04.030 23624387

[B37] Janeway C. A. Medzhitov R. (2002). Innate Immune Recognition. Annu. Rev. Immunol. 20, 197–216. doi: 10.1146/annurev.immumol.20.0830002.084359 11861602

[B38] Jin M. S. Kim S. E. Heo J. Y. Lee M. E. Kim H. M. Paik S.-G.. (2007). Crystal Structure of the TLR1-TLR2 Heterodimer Induced by Binding of a Tri-Acylated Lipopeptide. Cell 130, 1071–1082. doi: 10.1016/j.cell.2007.09.008 17889651

[B39] Josenhans C. Suerbaum S. (2002). The Role of Motility as a Virulence Factor in Bacteria. Int. J. Med. Microbiol. 291, 605–614. doi: 10.1078/1438-4221-00173 12008914

[B40] Kagan J. C. Su T. Horng T. Chow A. Akira S. Medzhitov R. (2008). TRAM Couples Endocytosis of Toll-Like Receptor 4 to the Induction of Interferon-β. Nat. Immunol. 9, 361–368. doi: 10.10.1038/ni1569 18297073 PMC4112825

[B41] Kang J. Y. Nan X. Jin M. S. Youn S.-J. Ryu Y. Mah S.. (2009). Recognition of Lipopeptide Patterns by Toll-Like Receptor 2-Toll-Like Receptor 6 Heterodimer. Immunity 31, 873–884. doi: 10.1016/j.immuni.2009.09.018 19931471

[B42] Kawahara K. Tsukano H. Watanabe H. Lindner B. Matsuura M. (2002). Modification of the Structure and Activity of Lipid A in Yersinia Pestis Lipopolysaccharide by Growth Temperature. Infect. Immun. 70, 4092–4098. doi: 10.1128/IAI.70.8.4092-4098.2002 12117916 PMC128165

[B43] Kawai T. Akira S. (2010). The Role of Pattern-Recognition Receptors in Innate Immunity: Update on Toll-Like Receptors. Nat. Immunol. 11, 373–384. doi: 10.1038/ni.1863 20404851

[B44] Kim J. S. Reuhs B.L. Rahman M.M. Ridley B. Carlson R.W. (1996). Separation of Bacterial Capsular and Lipopolysaccharides by Preparative Electrophoresis. Glycobiol. 6, 433–437. doi: 10.1093/glycob/6.4.433 8842707

[B45] Kitchens R. L. Thompson P. A. O’Keefe G. E. Munford R. S. (2000). Plasma Constituents Regulate LPS Binding to, and Release From, the Monocyte Cell Surface. J. Endotox. Res. 6, 477–482. doi: 10.1179/096805100101532450 11521074

[B46] Kovacs-Simon A. Titball R. W. Michell S. L. (2011). Lipoproteins of Bacterial Pathogens. Infect. Immun. 79, 548–561. doi: 10.1128/IAI.00682-10 20974828 PMC3028857

[B47] Kurokawa K. Ryu K.-H. Ichikawa R. Masuda A. Kim M.-S. Lee H.. (2012). Novel Bacterial Lipoprotein Structures Conserved in Low-GC Content Gram-Positive Bacteria Are Recognized by Toll-Like Receptor 2. J. Biol. Chem. 287, 13170–13181. doi: 10.1074/jbc.M111.292235 22303020 PMC3339964

[B48] Li G.-W. Burkhardt D. Gross C. Weissman J. S. (2014). Quantifying Absolute Protein Synthesis Rates Reveals Principles Underlying Allocation of Cellular Resources. Cell 157, 624–635. doi: 10.1016/j.cell.2014.02.033 24766808 PMC4006352

[B49] Logue C.-A. Peak I. R. Beacham I. R. (2009). Facile Construction of Unmarked Deletion Mutants in *Burkholderia pseudomallei* Using sacB Counter-Selection in Sucrose-Resistant and Sucrose-Sensitive Isolates. J. Microbiol. Methods 76, 320–323. doi: 10.1016/j.mimet.2008.12.007 19150470

[B50] Matsuura M. (2013). Structural Modifications of Bacterial Lipopolysaccharide That Facilitate Gram-Negative Bacteria Evasion of Host Innate Immunity. Front. Immunol. 4, 109. doi: 10.3389/fimmu.2013.00109 23745121 PMC3662973

[B51] Matsuura M. Takahashi H. Watanabe H. Saito S. Kawahara K. (2010). Immunomodulatory Effects of Yersinia Pestis Lipopolysaccharides on Human Monocytes. Clin. Vaccine Immunol. 17, 49–55. doi: 10.1128/CVI.00336-09 19889939 PMC2812085

[B52] McNab F. Mayer-Barber K. Sher A. Wack A. O’Garra A. (2015). Type I Interferons in Infectious Disease. Nat. Rev. Immunol. 15, 87–103. doi: 10.1038/nri3787 25614319 PMC7162685

[B53] Montminy S. W. Khan N. McGrath S. Walkowicz M. J. Sharp F. Conlon J. E.. (2006). Virulence Factors of *Yersinia pestis* Are Overcome by a Strong Lipopolysaccharide Response. Nat. Immunol. 7, 1066–1073. doi: 10.1038/ni.1386 16980981

[B54] Nakayama H. Kurokawa K. Lee B. L. (2012). Lipoproteins in Bacteria: Structures and Biosynthetic Pathways. FEBS J. 279, 4247–4268. doi: 10.1111/febs.12041 23094979

[B55] Nierman W. C. DeShazer D. Kim H. S. Tettelin H. Nelson K. E. Feldblyum T.. (2004). Structural Flexibility in the *Burkholderia mallei* Genome. Proc. Natl. Acad. Sci. 101, 14246–14251. doi: 10.1073/pnas.0403306101 15377793 PMC521142

[B56] Nithichanon A. Rinchai D. Gori A. Lassaux P. Peri C. Conchillio-Sole O.. (2015). Sequence –and Structural-Based Immunoreactive Epitope Discovery for *Burkholderia pseudomallei* Flagellin. PloS Negl. Trop. Dis. 9 (7), e0003917. doi: 10.1371/journal.pntd.0003917 26222657 PMC4519301

[B57] Okan N. A. Kasper D. L. (2013). The Atypical Lipopolysaccharide of *Francisella* . Carbohydr. Res. 378, 79–83. doi: 10.1016/j.carres.2013.06.015 23916469 PMC3776585

[B58] Papadopoulos J. S. Agarwala R. (2007). COBALT: Constraint-Based Alignment Tool for Multiple Protein Sequences. Bioinformatics 23, 1073–1079. doi: 10.1093/bioinformatics/btm076 17332019

[B59] Perry R. D. Fetherston J. D. (1997). *Yersinia pestis*-Etiologic Agent of Plague. Clin. Microbiol. Rev. 10, 35–66. doi: 10.1128/CMR.10.1.35 8993858 PMC172914

[B60] Poltorak A. He X. Smirnov I. Liu M.-Y. Huffel C. V. Du X.. (1998). Defective LPS Signaling in C3H/HeJ and C57BL/10ScCr Mice: Mutations in Tlr4 Gene. Science 282, 2085–2088. doi: 10.1126/science.282.5396.2085 9851930

[B61] Raetz C. R. H. Guan Z. Ingram B. O. Six D. A. Song F. Wang X.. (2009). Discovery of New Biosynthetic Pathways: The Lipid A Story. J. Lipid Res., S103–S108. doi: 10.1194/jlr.R800060-JI.R200 18974037 PMC2674688

[B62] Ramos H. C. Rumbo M. Sirard J.-C. (2004). Bacterial Flagellins: Mediators of Pathogenicity and Host Immune Responses in Mucosa. Trends Microbiol. 12, 509–517. doi: 10.1016/j.tim.2004.09.002 15488392

[B63] Rebeil R. Ernst R. K. Gowen B. B. Miller S. I. Hinnebusch B. J. (2004). Variation in Lipid A Structure in the Pathogenic Yersiniae. Mol. Microbiol. 52, 1363–1373. doi: 10.1111/j.1365-2958.2004.04059.x 15165239

[B64] Reckseidler-Zenteno S. L. DeVinney R. Woods D. E. (2005). The Capsular Polysaccharide of *Burkholderia pseudomallei* Contributes to Survival in Serum by Reducing Complement Factor C3b Deposition. Infect. Immun. 73, 1106–1115. doi: 10.1129/IAI.73.2.1106-1115.2005 15664954 PMC547107

[B65] Rowe H. M. Huntley J. F. (2015). From the Outside-in: The *Francisella tularensis* Envelope and Virulence. Front. Cell. Infect. Microbiol. 5, 94. doi: 10.3389/fcimb.2015.00094 26779445 PMC4688374

[B66] Russell A. D. (2003). Lethal Effects of Heat on Bacterial Physiology and Structure. Sci. Prog. 86, 115–137. doi: 10.3184/003685003783238699 12838607 PMC10368340

[B67] Schmid J. Sieber V. Rehm B. (2015). Bacterial Exopolysaccharides: Biosynthesis Pathways and Engineering Strategies. Front. Microbiol. 6, 496. doi: 10.3389/fmicb.2015.00496 26074894 PMC4443731

[B68] Sengyee S. Yoon S. H. Paksanont S. Yimthin T. Wuthiekanun V. Limmathurotsakul D.. (2018). Comprehensive Analysis of Clinical Burkholderia Pseudomallei Isolates Demonstrates Conservation of Unique Lipid A Structure and TLR4-Dependent Innate Immune Activation. PloS Negl. Trop. Dis. 12 (2), e0006287. doi: 10.1371/journal.pntd.0006287 29474381 PMC5842036

[B69] Shea A. A. Benhards R. C. Cote C. K. Chase C. J. Koehler J. W. Klimko C. P.. (2017). Two Stable Variants of *Burkholderia Pseudomallei* Strain MSHR5848 Express Broadly Divergent *In Vitro* Phenotypes Associated With Their Virulence Differences. PloS One 12 (2), e0171363. doi: 10.1371/journal.pone.0171363 28187198 PMC5302386

[B70] Shimazu R. Akashi S. Ogata H. Nagai Y. Fukudome K. Miyake K.. (1999). MD-2, a Molecule That Confers Lipopolysaccharide Responsiveness on Toll-Lke Receptor 4. J. Exp. Med. 189, 1777–1782. doi: 10.1084/jem.189.11.1777 10359581 PMC2193086

[B71] Simon R. Quandt J. Klipp W. (1989). New Derivatives of Transposon Tn5 Suitable for Mobilization of Replicons, Generation of Operon Fusions and Induction of Gene in Gram-Negative Bacteria. Gene 80, 161–169. doi: 10.1016/0378-1119(89)90262-x.5 2551782

[B72] Smith K. D. Andersen-Nissen E. Hayashi F. Strobe K. Bergman M. A. Barrett S. L. R.. (2003). Toll-Like Receptor 5 Recognizes a Conserved Site on Flagellin Required for Protofilament Formation and Bacterial Motility. Nat. Immunol. 4, 1247–1253. doi: 10.1038/ni1011 14625549

[B73] Takeuchi O. Akira S. (2010). Pattern Recognition Receptors and Inflammation. Cell 140, 805–820. 20303872 10.1016/j.cell.2010.01.022

[B74] Takeuchi O. Sato S. Horiuchi T. Hoshino K. Takeda K. Dong Z.. (2002). Role of Toll-Like Receptor 1 in Mediating Immune Response to Microbial Lipoproteins. J. Immunol. 169, 10–14. doi: 10.4049/jimmunol.169.1.10 12077222

[B75] Tesh V. L. Duncan R. L. Morrison J. D.C. (1986). The Interaction of *Escherichia coli* With Normal Human Serum: The Kinetics of Serum-Mediated Lipopolysaccharide Release and Its Dissociation From Bacterial Killing. J. Immunol. 137, 1329–1335. doi: 10.1016/0882-4010(88)90068-X 3525676

[B76] Thakran S. Li H. Lavine C. L. Miller M. A. Bina J. E. Bina X. R.. (2008). Identification of *Francisella tularensis* Lipoproteins That Stimulate the Toll-Like Receptor (TLR) 2/TLR1 Heterodimer. J. Biol. Chem. 238, 3751–3760. doi: 10.1074/jbc.M706854200 18079113

[B77] Trevino S. R. Klimko C. P. Reed M. C. Aponte-Cuadrado M. J. Hunter M. Shoe J. L.. (2018). Disease Progression in Mice Exposed to Low-Doses of Aerosolized Clinical Isolates of Burkholderia Pseudomallei. PloS One 13 (11), e0208277. doi: 10.1371/journal.pone.0208277 30500862 PMC6267979

[B78] Verma A. K. Saminathan M. Neha Tiwari R. Dhama K. Singh S. V. (2014). Glanders-A Re-Emerging Zoonotic Disease: A Review. J. Biol. Sci. 14, 38–51. doi: 10.3923/jbs.2014.38.51

[B79] Vesy C. J. Kitchens R. L. Wolfbauer G. Albers J. J. Munford R. S. (2000). Lipopolysaccharide-Binding Protein and Phospholipid Transfer Protein Release Lipopolysaccharides From Gram-Negative Bacterial Membranes. Infect. Immun. 68, 2410–2417. doi: 10.1128/IAI.68.5.2410-2417.2000 10768924 PMC97439

[B80] Wang Q. McLoughlin R. M. Cobb B. A. Charrel-Dennis M. Zaleski K. L. Golenbock D.. (2006). A Bacterial Carbohydrate Links Innate and Adaptive Responses Through Toll-Like Receptor 2. J. Exp. Med. 203, 2853–2863. doi: 10.1084/jem.20062008 17178920 PMC2118167

[B81] Wang X. Ribeiro A. A. Guan Z. McGrath S. C. Cotter R. J. Raetz R. H. (2006). Structure and Biosynthesis of Free Lipid A Molecules That Replace Lipopolysaccharide in *Francisella novicida* . Biochem 45, 14427–14440. doi: 10.1021/bi061767s 17128982 PMC2569856

[B82] Warawa J. M. Long D. Rosenke R. Gardner D. Gherardini F. C. (2009). Role for the *Burkholderia Pseudomallei* Capsular Polysaccharide Encoded by the Wcb Operon in Acute Disseminated Melioidosis. Infect. Immun. 77, 5252–5261. doi: 10.1128/IAI.00824-09 19752033 PMC2786491

[B83] West T. E. Chantratita N. Chierakul W. Limmathurotsakul D. Wuthiekanun V. Myers N. D.. (2013). Impaired TLR5 Functionality Is Associated With Survival in Melioidosis. J. Immunol. 190, 3373–3379. doi: 10.4049/jimmunol.1202974 23447684 PMC3607401

[B84] West T. E. Ernst R. K. Jansson-Hutson M. J. Skerrett S. J. (2008). Activation of Toll-Like Receptors by *Burkholderia pseudomallei* . BMC Immunol. 9:46. doi: 10.1186/1471-2172-9-46 18691413 PMC2527550

[B85] Whitfield C. (2006). Biosynthesis and Assembly of Capsular Polysaccharides in *Escherichia coli* . Annu. Rev. Biochem. 75, 39–68. doi: 10.1146/annurev.biochem.75.103004.142545 16756484

[B86] Whitfield C. Trent M. S. (2014). Biosynthesis and Export of Bacterial Lipopolysaccharides. Annu. Rev. Biochem. 83, 99–128. doi: 10.1146/annurev-biochem-060713-035600 24580642

[B87] Whitlock G. C. Estes D. M. Torres A. G. (2007). Glanders: Off to the Races With *Burkholderia mallei* . FEMS Microbiol. Lett. 277, 115–122. doi: 10.1111/j.1574-6968.2007.00949.x 18031330

[B88] Whitney J. C. Howell P. L. (2013). Synthase-Dependent Exopolysaccharide Secretion in Gram-Negative Bacteria. Trends Microbiol. 21, 63–72. doi: 10.1016/j.tim.201210.001 23117123 PMC4113494

[B89] Wiersinga W. J. Currie B. J. Peacock S. J. (2012). Melioidosis. N. Engl. J. Med. 367, 1035–1044. doi: 10.1056/NEJMraI204699 22970946

[B90] Wiersinga W. J. Virk H. S. Torres A. G. Currie B. J. Peacock S. J. Dance D. A. B.. (2018). Melioidosis. Nat. Rev. Dis. Primers 4, 17107. doi: 10.1038/nrdp.2017.107 29388572 PMC6456913

[B91] Wilson M. M. Bernstein H. D. (2016). Surface-Exposed Lipoproteins: An Emerging Secretion Phenomenon in Gram-Negative Bacteria. Trends Microbiol. 24, 198–208. doi: 10.1016/j.tim.2015.11.006 26711681 PMC9373711

[B92] Woodman M. E. Worth R. G. Wooten R. M. (2012). Capsule Influences the Deposition of Critical Complement C3 Levels Required for the Killing of *Burkholderia pseudomallei via* NADH-Oxidase Induction by Human Neutrophils. PloS One 7 (12), e52276. doi: 10.1371/journal.pone.0052276 23251706 PMC3522640

[B93] Yarovinsky F. Zhang D. Andersen J. F. Bannenberg G. L. Serhan C. N. Hayden M. S.. (2005). TLR11 Activation of Dendritic Cells by a Protozoan Profiling-Like Protein. Science 308, 1626–1629. doi: 10.1126/science.1109893 15860593

[B94] Yi E. C. Hackett M. (2000). Rapid Isolation Method for Lipopolysaccharide and Lipid A From Gram-Negative Bacteria. Analyst 125, 651–656. doi: 10.1039/b000368i 10892021

[B95] Yonekura K. Maki-Yonekura S. Namba K. (2003). Complete Atomic Model of the Bacterial Flagellar Filament by Electron Cryomicroscopy. Nature 424, 643–650. doi: 10.1038/nature01830 12904785

[B96] Yoon S. Kurnasov O. Natarajan V. Hong M. Budkov A. V. Ostermann A. L.. (2012). Structural Basis of TLR5-Flagellin Recognition and Signaling. Science 335, 859–864. doi: 10.1126/science.1215584 22344444 PMC3406927

[B97] Zhang S. Feng S.-H. Li B. Kim H.-Y. Rodriguez J. Tsai S.. (2011). *In Vitro* and *In Vivo* Studies of Monoclonal Antibodies With Prominent Bactericidal Activity Against *Burkholderia pseudomallei* and *Burkholderia mallei* . Clin. Vaccine Immumol. 18, 825–834. doi: 10.1128/CVI.00533-10 PMC3122519 21450976

[B98] Zhang H. Niesel D. W. Peterson J. W. Klimpel G. R. (1998). Lipoprotein Release by Bacteria: Potential Factor in Bacterial Pathogenesis. Infect. Immun. 66, 5196–5201. doi: 10.1128/IAI.66.11.5196-5204.1998 9784522 PMC108648

[B99] Zhang D. Zhang G. Hayden M. S. Greenblatt M. B. Bussey C. Flavell R. A.. (2004). A Toll-Like Receptor That Prevents Infection by Uropathogenic Bacteria. Science 303, 1522–1526. doi: 10.1126/science.1094351 15001781

