# Supplementary figures and images for: Activation of Toll-Like Receptors by Live Gram-Negative Bacterial Pathogens Reveals Mitigation of TLR4 Responses and Activation of TLR5 by Flagella
Source: Front Cell Infect Microbiol. 2021 Nov 23;11:745325. doi: 10.3389/fcimb.2021.745325 (PMC8650638; doi:10.3389/fcimb.2021.745325)

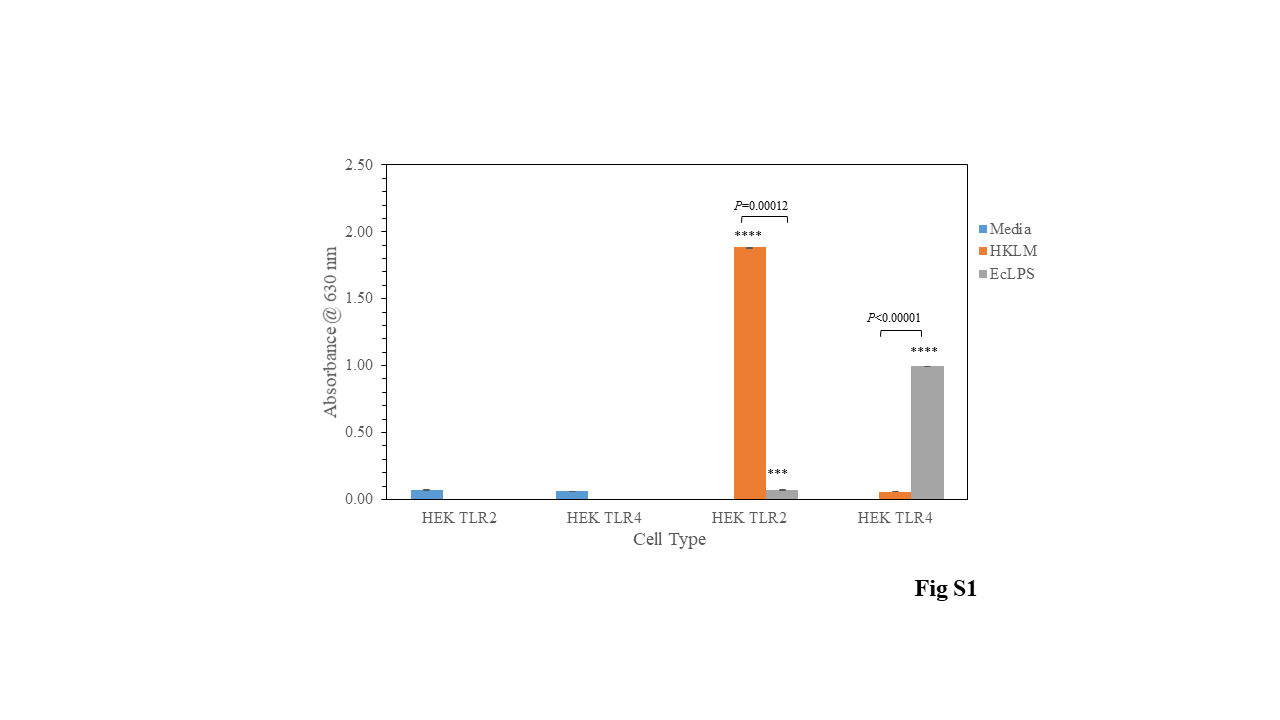

Supplement: Supplementary Figure 1 — HEK293-TLR2 and HEK293-TLR4 cells show high specificity to their specific agonist. HEK TLR2 and HEK TLR4 cells were grown and harvested as described in the Methods section. The cells were inoculated into 96-well plates in triplicate and incubated with their respective agonist, HKLM (2 x 10^6^ cells) for HEK TLR2 cells or Ec LPS (0.2 ng) for HEK TLR4 cells. Another set of TLR2 cells were incubated with Ec LPS and another set of TLR4 cells were incubated with HKLM. After overnight incubation at 37°C with 5% CO2 the plates were read at 630 nm. Significant differences between the agonist on the specific cell line are shown above the data. Results of TLR activation are presented as geometric mean with standard error of the mean. Significant values compared to media control are shown: ***P ≤ 0.001; ****P ≤ 0.0001. [file Image_1.tif]
